# Endovascular ablation of the right greater splanchnic nerve for the management of heart failure with preserved ejection fraction: a systematic review and meta-analysis

**DOI:** 10.1186/s43044-026-00748-1

**Published:** 2026-06-08

**Authors:** Hashim Manea, Mostafa A. Khalifa, Mustafa A. Shahrori, Aya Ahmed Shimal, Ahmad Omar Saleh, Omnia Samy El-Sayed, Muhammad Bashir, Elian Khalafalla Awadalla, Alaa R. AL-Ihribat, Fathimathul Henna, Mohammedbaqer Al-Ghuraibawi, Mohammad Amjad Shahrori

**Affiliations:** 1 University of Jamestown, Jamestown, ND USA; 2 Cairo University , Cairo, Egypt; 3 An-Najah National University, Nablus, Palestine; 4 University of Baghdad, Baghdad, Iraq; 5 University of Jordan, Amman, Jordan; 6 Zagazig University , Zagazig, Egypt; 7 Fazaia Ruth Pfau Medical College , Karachi, Pakistan; 8 University of Khartoum, Khartoum, Sudan; 9 Palestine Polytechnic University, Hebron, Palestine; 10 Dubai Medical College for Girls, Dubai, United Arab Emirates; 11 University of Warith Al-Anbiyaa, Karbala, Iraq

**Keywords:** Meta-analysis, Endovascular ablation, Right greater splanchnic nerve (GSN), Heart failure with preserved ejection fraction (HFpEF), Efficacy and safety, Estimated glomerular filtration rate (eGFR), Cardiovascular health, Blood pressure, Randomized controlled trials

## Abstract

**Background:**

Heart failure with preserved ejection fraction (HFpEF) remains difficult to manage because conventional therapies often provide limited symptom relief and do not adequately address exercise-related congestion. Right greater splanchnic nerve (GSN) ablation has emerged as a potential therapeutic approach by modulating splanchnic venous capacitance. However, current evidence is limited and includes both uncontrolled and randomized studies.

**Methods:**

We performed a systematic review and meta-analysis of studies evaluating right GSN ablation in HFpEF. Searches were conducted in PubMed, Scopus, and Embase in December 2024. Randomized and non-randomized studies were included. Continuous outcomes were pooled as mean differences (MDs) with 95% confidence intervals (CIs). Sensitivity analyses excluding the randomized controlled trial (RCT) were performed for selected outcomes. Analyses based on uncontrolled studies and converted median/interquartile range data were considered exploratory.

**Results:**

The most consistent findings were observed in functional and exercise-related hemodynamic outcomes. Six-minute walk distance improved at both 6 and 12 months, and KCCQ overall score showed improvement, although with substantial heterogeneity. Provocative hemodynamic measures, including 20 W and peak pulmonary capillary wedge pressure, were reduced at 1 month, whereas resting hemodynamic measures showed no clear pooled benefit. NT-proBNP, renal indices, blood pressure, heart rate, and left ventricular ejection fraction were largely unchanged. Overall, randomized sham-controlled evidence did not demonstrate a clear comparative benefit.

**Conclusions:**

Right GSN ablation may improve selected exercise-related hemodynamic, functional, and patient-reported outcomes in HFpEF, but the current evidence base is driven mainly by small uncontrolled studies. Randomized evidence remains neutral, and further sham-controlled trials are required.

**Supplementary Information:**

The online version contains supplementary material available at 10.1186/s43044-026-00748-1.

## Introduction

Heart failure with preserved ejection fraction (HFpEF) now accounts for nearly half of all heart failure cases. It is a burden marked by frequent hospitalizations, persistent congestion, and a significant decline in exercise capacity and quality of life [1]. As our population ages and the prevalence of obesity, hypertension, diabetes, atrial fibrillation, and chronic kidney disease grows, so does the impact of HFpEF [1, 2]. Even with the success of SGLT2 inhibitors in improving the treatment landscape, many patients remain symptomatic. This persistent exercise intolerance and congestion highlight a clear need for new therapies that target mechanisms current treatments simply do not reach [3–6].

A major part of the problem in HFpEF is an abnormal hemodynamic reserve. Exercise or stress causes a rise in cardiac filling pressures and pulmonary congestion [2, 7, 8]. During these moments of stress, the sympathetic nervous system activates and narrows the splanchnic venous compartment, the body’s main abdominal blood reservoir, forcing blood upward into the thoracic circulation [9, 10]. For a patient with a stiff, noncompliant left ventricle, this sudden shift in volume drives up pulmonary capillary wedge pressure (PCWP), triggering dyspnea and worsening congestion [7, 9, 10]. Because the greater splanchnic nerve (GSN) is a key regulator of this reservoir, it has become a biologically plausible therapeutic target [9, 10].

Interest in GSN modulation is growing because it tackles a mechanism that standard HFpEF drugs do not directly address: the redistribution of blood volume during physical exertion [3, 9, 10]. By interrupting right-sided GSN signals through surgical or endovascular procedures, splanchnic capacitance may increase, thereby blunting pressure spikes during stress and potentially improving symptoms and function [10–14]. Early studies appeared to support this concept, showing favorable changes in exercise hemodynamics, functional capacity, and patient-reported outcomes after right GSN ablation [11–15].

However, the clinical evidence remains limited and must be interpreted cautiously. While early uncontrolled studies suggested potential benefit, the randomized sham-controlled REBALANCE-HF trial did not show a significant reduction in exercise PCWP or clear overall clinical benefit in a broad HFpEF population [16]. These mixed findings suggest that right GSN ablation is unlikely to be a one-size-fits-all solution and that any benefit may depend on identifying specific responder phenotypes [16, 17].

Given these conflicting findings, a systematic assessment of the evidence is needed, with clear distinction between exploratory uncontrolled data and randomized comparative results. Therefore, this systematic review and meta-analysis evaluates the safety and the clinical, functional, hemodynamic, biomarker, renal, and echocardiographic effects of right GSN ablation in HFpEF. Our aim is to clarify the current scope and limitations of the evidence and to identify key priorities for future research.

## Methods

### Literature search

A systematic literature search was conducted in December 2024 using PubMed, Scopus, and Embase to identify studies evaluating right greater splanchnic nerve ablation in patients with HFpEF. The search yielded 55 records in total. Google Scholar and ScienceDirect were searched; however, no studies were extracted from them. The full reproducible search strings, database-specific strategies, and exact search dates are provided in the Supplementary Document. Conference abstracts were excluded because of limited methodological detail, incomplete outcome reporting, and the inability to reliably assess overlap with subsequent full-text publications; this may have introduced publication bias and should be considered when interpreting the findings.

### Study selection criteria and screening processe 

All retrieved records were imported into Rayyan for screening and review management, and duplicates were removed. Two authors independently screened titles and abstracts according to predefined inclusion and exclusion criteria, with disagreements resolved through discussion or consultation with a third reviewer. Fulltext articles of potentially eligible studies were then assessed for final inclusion. Eligible studies included randomized and non-randomized trials, cohort studies, case series, and case reports evaluating right greater splanchnic nerve ablation in patients with HFpEF. Excluded records comprised systematic reviews, letters to the editor, conference abstracts, conference papers, surveys, animal studies, and other non-relevant designs. During full-text assessment, particular attention was paid to identifying overlapping or serial publications from the same cohort or study program, and such reports were consolidated during synthesis to avoid double-counting.

### Data extraction

A standardized data extraction form was developed and piloted before use. Two reviewers independently extracted data, and discrepancies were resolved through discussion or consultation with a third reviewer. Extraction was guided by the PICO framework and included study characteristics, participant and intervention details, follow-up duration, and reported outcomes. Outcomes of interest were categorized a priori as primary and secondary outcomes. Primary outcomes included functional capacity, hemodynamic parameters, and patient-reported health status, specifically 6-minute walk test (6MWT), pulmonary capillary wedge pressure (PCWP), and Kansas City Cardiomyopathy Questionnaire (KCCQ) overall score. Secondary outcomes included biomarker and renal measures, echocardiographic and cardiac function parameters, additional hemodynamic measures, and safety or clinical event outcomes. For quantitative synthesis, continuous outcomes were extracted as reported, including baseline and follow-up values, change scores, and measures of dispersion where available and overlapping reports were cross-checked to minimize double-counting. When outcomes were reported as median and interquartile range (IQR) rather than mean and standard deviation (SD), approximate mean and SD values were estimated using established conversion approaches when quantitative synthesis was considered necessary. These converted analyses were treated as exploratory and interpreted cautiously. The full data extraction form and detailed extracted variables are provided in the Supplementary Material.

.

### Risk-of-bias assessment 

Methodological quality was independently assessed by two reviewers using the Cochrane RoB 2.0 tool for randomized studies and ROBINS-I for non-randomized studies. These tools were selected because the included evidence comprised both randomized and non-randomized designs, requiring design-specific risk-of-bias assessment. RoB 2.0 evaluates bias in randomized trials across key domains related to randomization, deviations from intended interventions, missing outcome data, outcome measurement, and selective reporting, whereas ROBINS-I is designed for non-randomized studies and assesses bias arising from confounding, participant selection, intervention classification, deviations from intended interventions, missing data, outcome measurement, and selection of reported results. Disagreements were resolved through discussion or consultation with a third reviewer. Studies were classified according to standard tool guidance, and risk-of-bias findings were considered when interpreting the results.

### Data analysis and heterogeneity

Quantitative synthesis was performed using RStudio. Analyses were performed using within-arm pre–post changes for both randomized and non-randomized studies. Outcomes were analyzed at 1, 3, 6, and 12 months of follow-up, where available. Continuous outcomes were summarized as mean differences (MDs) with corresponding 95% confidence intervals (CIs). When pooling was feasible, a random-effects model was used because of the small number of eligible studies per outcome and timepoint, as well as the expected clinical and methodological heterogeneity across the included studies. For outcomes reported in both randomized and non-randomized studies, pooled analyses including all available studies were conducted first, followed by sensitivity analyses excluding the randomized controlled trial to assess the robustness of findings within predominantly uncontrolled evidence. Statistical heterogeneity was assessed using the I² statistic and Cochrane’s Q test. I² values of < 25%, 25%–50%, 50%–75% and > 75% were interpreted as low, moderate, substantial and high heterogeneity, respectively. Given the small number of eligible studies per outcome and timepoint, and the clinical and methodological heterogeneity of the evidence base, pooled findings were interpreted cautiously. Analyses based on uncontrolled studies and those requiring conversion of median and interquartile range to approximate mean and standard deviation were considered exploratory, where appropriate.

## Results

### Study selection

**Fig. 1:**
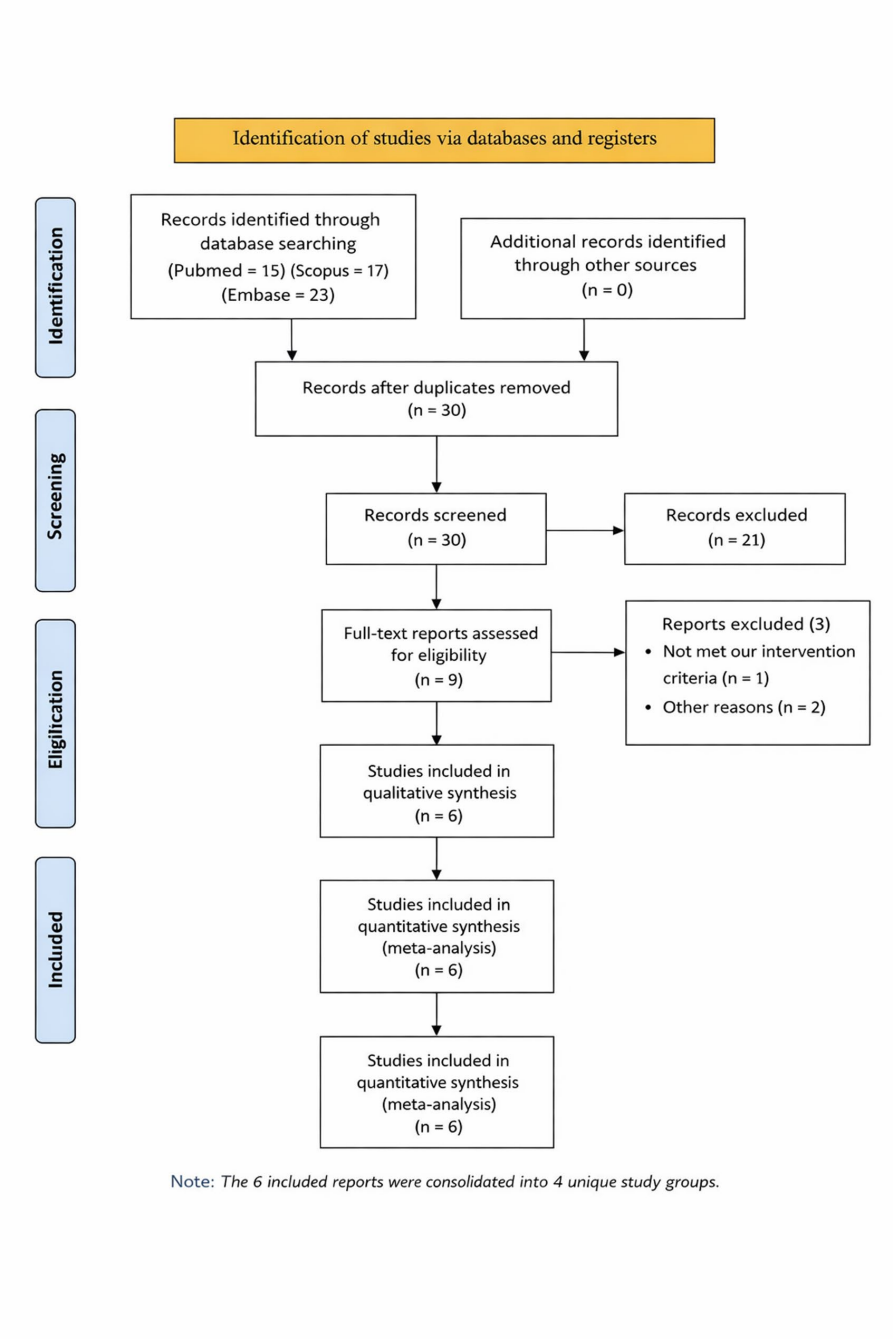
This chart illustrates the study’s identification and screening process

The study selection process is illustrated in the PRISMA flow diagram (Fig. 1). A total of 55 records were identified through database searching, including 15 from PubMed, 17 from Scopus, and 23 from Embase. No additional records were identified through other sources. After duplicate removal, 30 unique records remained for title and abstract screening. Of these, 21 records were excluded. Nine full-text reports were assessed for eligibility, of which three were excluded. Ultimately, six studies were included in the qualitative analysis and four of them underwent quantitative analysis (meta-analysis). Because some included reports represented serial or overlapping publications from the same study program, these were handled cautiously during synthesis to avoid double-counting of non-independent data.

### Baseline characteristics

Baseline characteristics are presented at the report level to describe the included literature. Across the six included reports, a total of 116 treated participants were described. However, some reports represented overlapping or serial publications from the same study program. Specifically, Gajewski et al. was a subset analysis of the original Málek et al. surgical cohort, and the 18-patient REBALANCE-HF roll-in report was subsequently incorporated into the 26-patient REBALANCE-HF lead-in report. After accounting for these overlaps, the six reports corresponded to four unique study groups comprising 91 unique treated participants. Participants were generally older adults, with reported baseline ages ranging from 67 ± 11 years to 74 ± 9 years in studies reporting mean ± SD, and 72 (64–79) years in the randomized SAVM arm and 72 (66–77) years in the 26-patient open-label lead-in cohort in studies reporting median (IQR). Accordingly, the baseline tables remain descriptive report-level summaries, whereas the quantitative synthesis was performed using the four non-overlapping study groups to avoid double-counting. See Tables 1, 2 and 3.

#### Patients characteristics

**Table 1:** Baseline patient characteristics of the included reports

Study ID	Study Type	Sample Size	Age (Mean/SD)	Gender (M/F)	BMI (Mean/SD)
Málek − 2021	Single-arm	10	70/3	M5/F5	31.67/5.16
Fudim − 2022	Single-arm	11	70/8	M3/F8	31.73/2.71
Fudim − 2024	Randomized Clinical Trial	44	71.66/11.49	(20/24)	32.56/6.74
Fudim − 2022	Single-arm	18	74/9	M4/F14	33.36/7.72
Gajewski − 2022	Single-arm	7	67 ± 11	M5/F2	31.33/5.51
Fudim − 2024	Single-arm	26	71.66/9.41	M7/F19	35.26/10.27

BMI, body mass index; RCT, randomized clinical trial; SAVM, splanchnic ablation for volume management

#### Comorbidities (N of Event)

**Table 2:** Baseline comorbidities across included reports

Study ID	Hypertension (*N* of Event)	Diabetes (*N* of Event)	MI (*N* of Event)	Coronary Disease (*N* of Event)	Atrial Fibrillation (*N* of Event)
Málek − 2021	8	6	4	6	9
Fudim − 2022	10	5	2	2	2
Fudim − 2024	37	18	15	24	36
Fudim − 2022	6	2	0	3	4
Gajewski − 2022	5	3	3	4	6
Fudim − 2024	21	8	5	15	18

HTN, hypertension; DM, diabetes mellitus; MI, myocardial infarction; CAD, coronary artery disease; AF, atrial fibrillation; NR, not reported

#### Medications (N of Event)

**Table 3:** Baseline medication use across included reports

Study ID	Loop Diuretic (*N* of Event)	Beta-blocker (*N* of Event)	ACE or ARB (*N* of Event)	CCB (*N* of Event)	MRA (*N* of Event)
Málek − 2021	10	8	8	2	6
Fudim − 2022	11	10	11	8	2
Fudim − 2024	24	NA	NA	NA	NA
Fudim − 2022	6	4	2	3	-
Gajewski − 2022	7	6	6	2	6
Fudim − 2024	18	NA	NA	NA	NA

ACEi/ARB, angiotensin-converting enzyme inhibitor/angiotensin receptor blocker; CCB, calcium channel blocker; MRA, mineralocorticoid receptor antagonist; NR, not reported

### Risk of bias

**Fig. 2:**
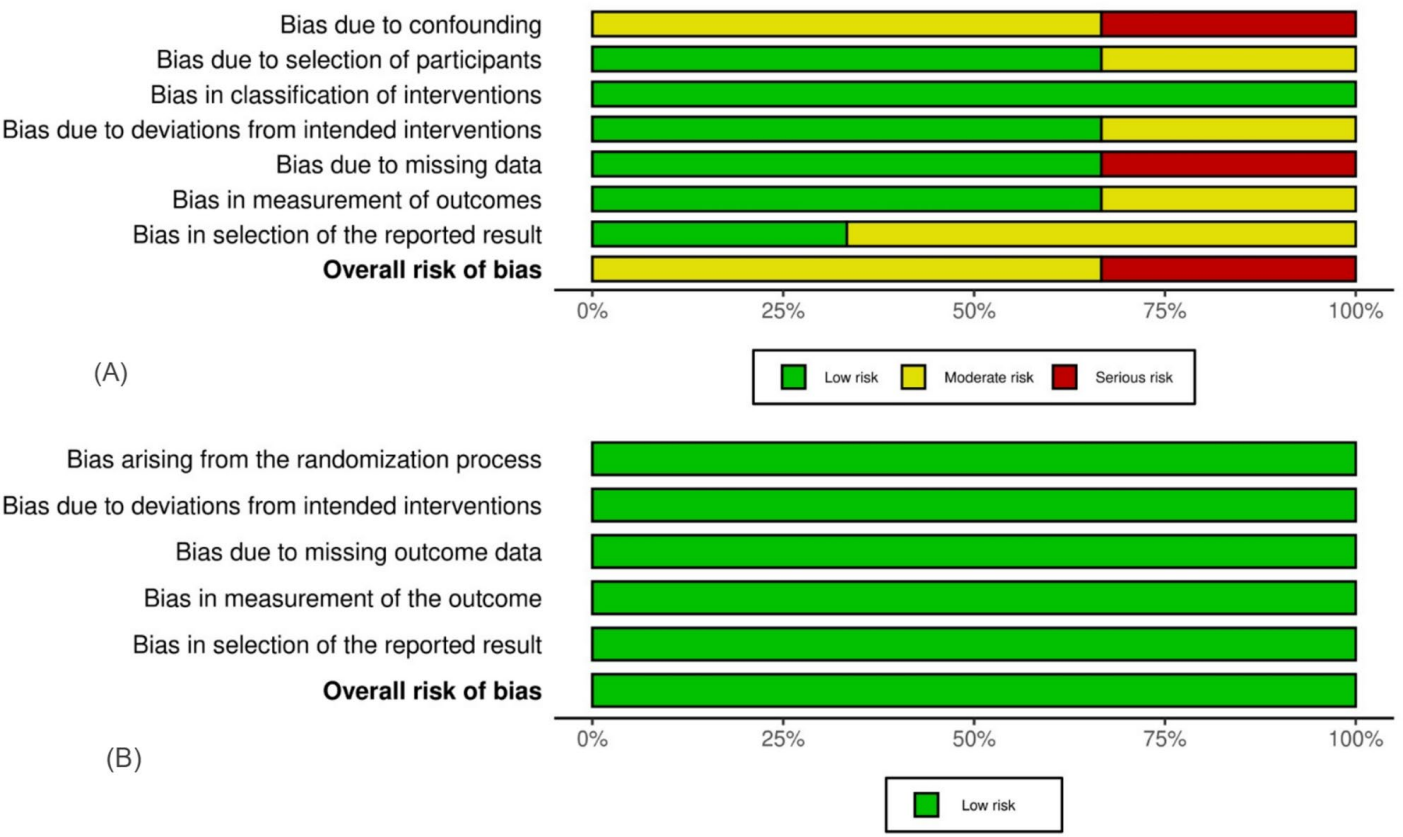
Assessment of risk of bias in the included studies. **A**: ROBINS-1 evaluation of observational studies. The panel presents a schematic representation of risks (low=green, moderate=yellow, and serious = red) for specific types of biases of each study in the review. **B**: ROB-2 evaluation of RCTs. The panel presents a schematic representation of risks (low=green) for specific types of biases of the study included in the review

Risk of bias was assessed at the report level using ROBINS-I for non-randomized reports and RoB 2.0 for the randomized trial. Among the five non-randomized reports, two were judged to have moderate risk of bias and one was judged to have serious risk of bias overall (Fig. 2A), with the principal concerns relating to confounding, selection of the reported result, and, in some cases, missing outcome data. These findings indicate that the favorable signals observed in the uncontrolled studies should be interpreted cautiously, as much of the non-randomized evidence base was derived from small reports with moderate-to-serious risk of bias. In contrast, the randomized trial by Fudim et al. 2024 was judged to be at low risk of bias across all RoB 2.0 domains (Fig. 2B), although randomized comparative evidence remains limited to a single trial.

### Functional and patient-reported outcomes

#### NYHA class distribution across follow-up time points

**Fig. 3:**
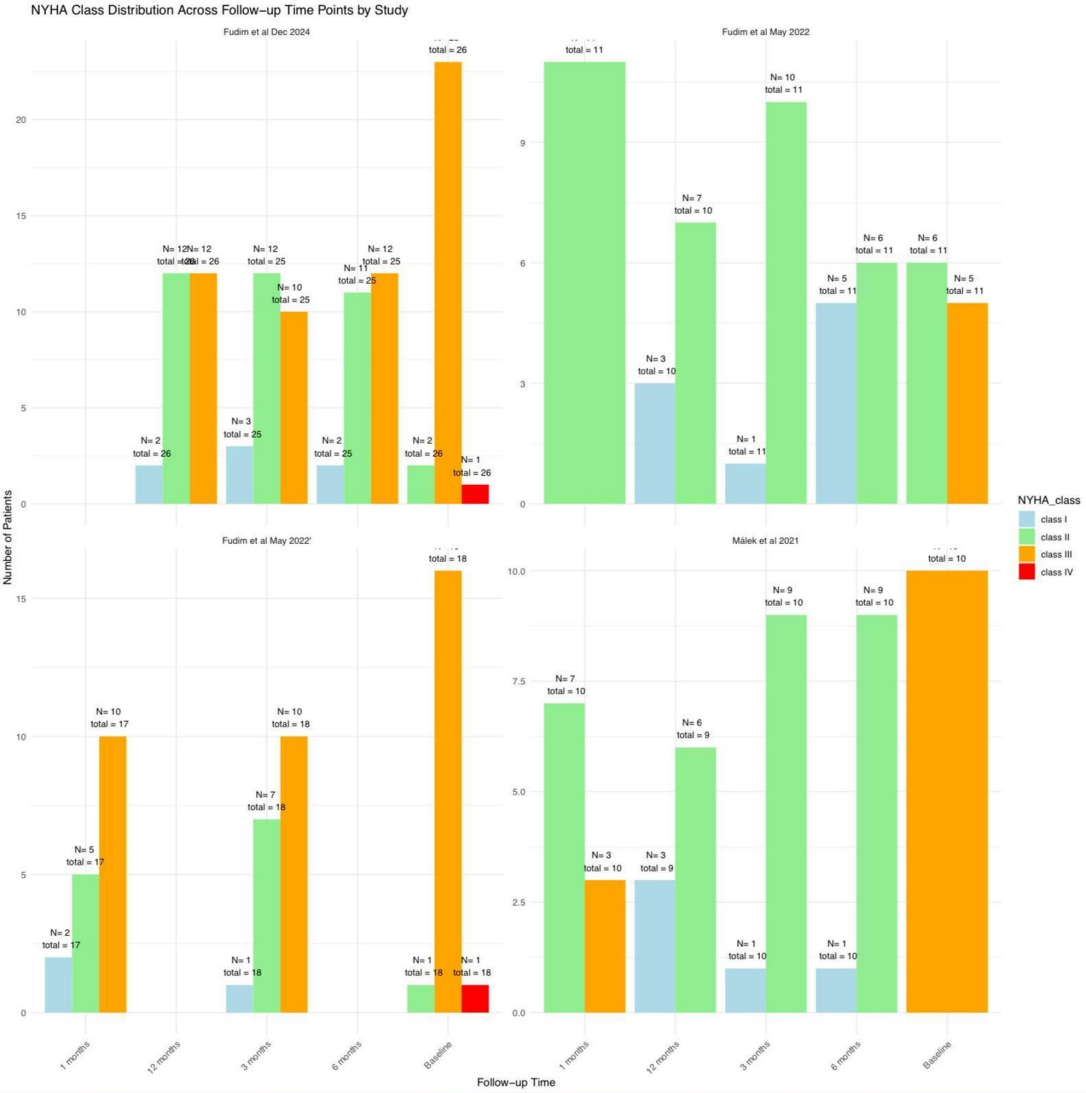
The chart illustrates the effect of endovascular ablation of the right greater splanchnic nerve on patients’ functional status, as measured by changes in the NYHA class over time, across different studies

Figure 3 presents a descriptive report-level summary of NYHA functional class across follow-up timepoints for the four included reports displayed in the figure. Where NYHA class was reported as percentages, the displayed counts were derived from the published percentages and corresponding sample sizes. Baseline symptom burden was generally high, with most patients in NYHA class III in the available uncontrolled cohorts, and follow-up data suggested a shift toward lower NYHA classes over time. Because NYHA was not reported in a fully uniform class-by-class format across studies, and because some displayed reports were overlapping publications from the same study program, Fig. 3 should be interpreted as a descriptive visualization only rather than a formal pooled comparative analysis (Fig. 3). In the randomized trial, NYHA improvement occurred in both the SAVM and sham groups at 3, 6, and 12 months, but no statistically significant between-group difference was observed, indicating that comparative efficacy for this endpoint remains unproven.

**Table 4:** Descriptive summary of NYHA functional class across follow-up in the included studies

Study shown in figure	Main NYHA pattern
Fudim et al. Dec 2024	Improvement over follow-up, mainly shift from NYHA III toward NYHA II
Fudim et al. May 2022 (*n* = 11)	Improvement over follow-up
Fudim et al. May 2022 (*n* = 17–18)	Early improvement over follow-up
Málek et al. 2021	Improvement over follow-up, mainly shift toward NYHA II

Table 4. This table provides a descriptive summary of the NYHA class figure and does not represent a pooled comparative analysis. Overall, uncontrolled studies suggested symptomatic improvement over time, but randomized evidence did not confirm a clear comparative benefit. Abbreviation: NYHA, New York Heart Association.

#### 6 minutes’ walk test

**Fig. 4:**
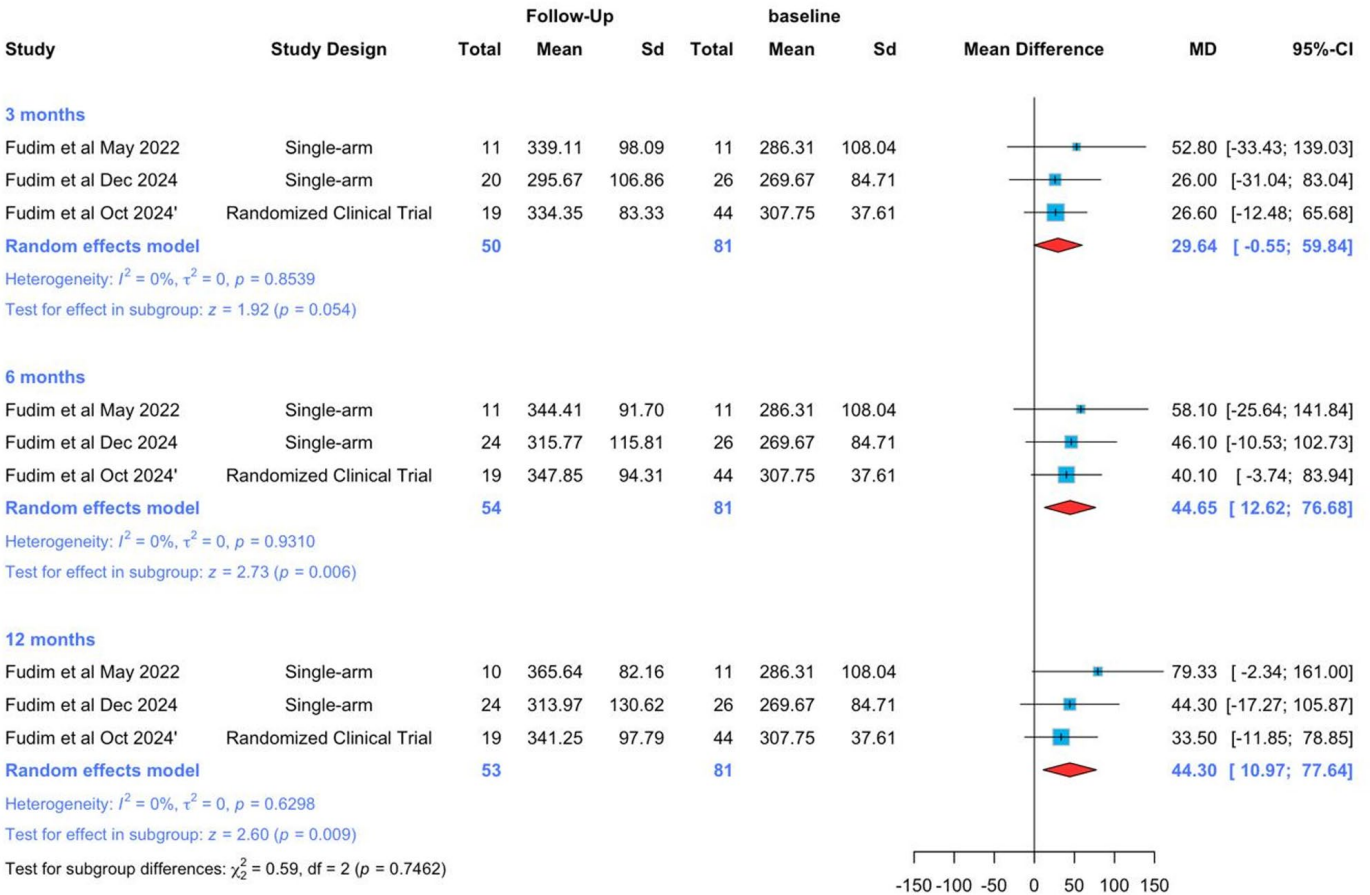
Forest plot of pooled effects for 6-minute walk test (6MWT) distance at 3, 6, and 12 months including all available studies

**Fig. 5:**
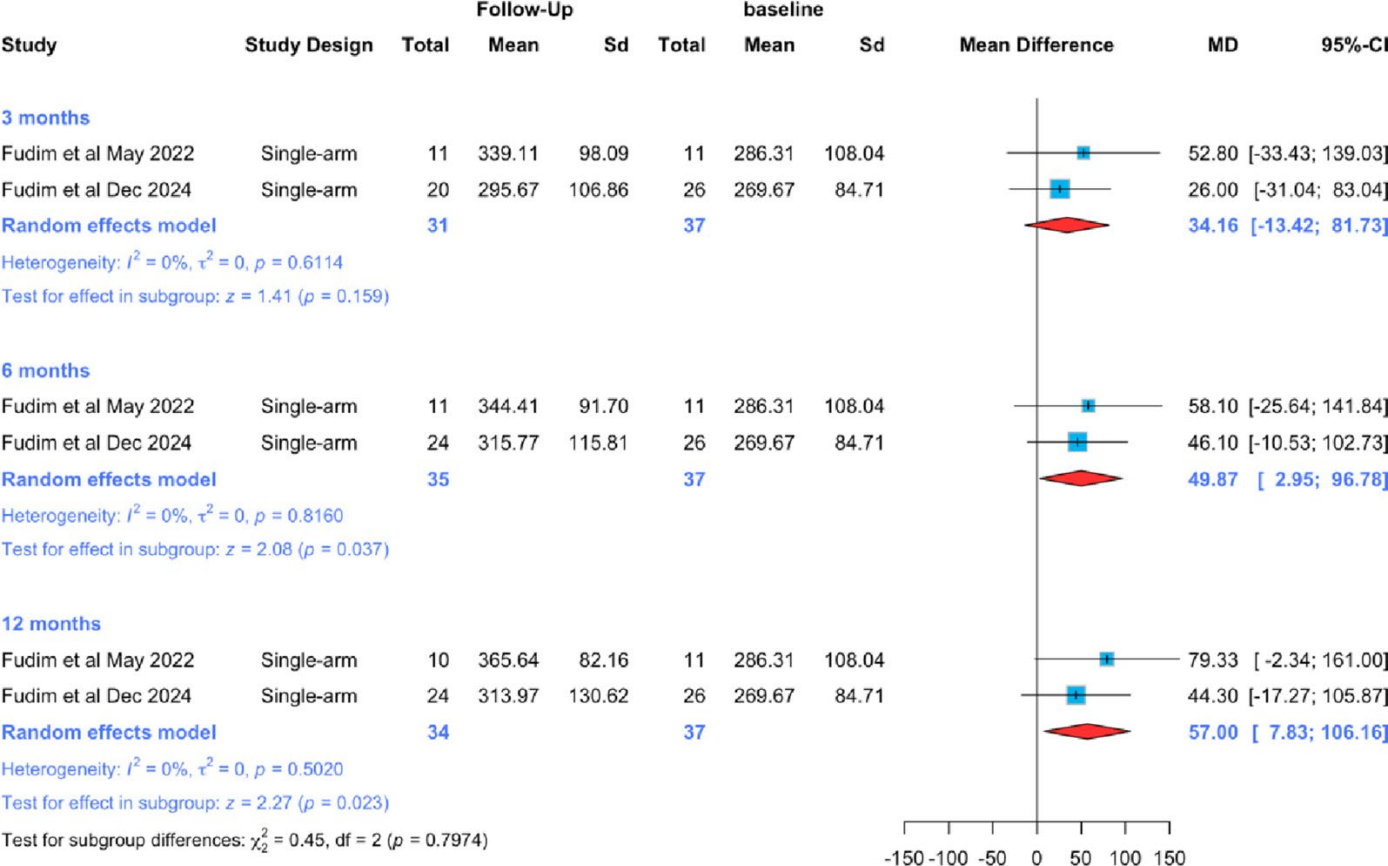
Sensitivity-analysis forest plot of pooled effects for 6-minute walk test (6MWT) distance at 3, 6, and 12 months after excluding the randomized controlled trial

When all available studies were included, pooled analysis showed improvement in 6MWT distance at all assessed follow-up timepoints, reaching statistical significance at 6 months and 12 months. The pooled mean difference was 29.64 m (95% CI − 0.55 to 59.84) at 3 months, 44.65 m (95% CI 12.62 to 76.68) at 6 months, and 44.30 m (95% CI 10.97 to 77.64) at 12 months, with no observed heterogeneity at any timepoint (I² = 0%). In sensitivity analysis excluding the randomized controlled trial, the direction of effect remained unchanged. The pooled mean difference was 34.16 m (95% CI − 13.42 to 81.73) at 3 months, 49.87 m (95% CI 2.95 to 96.78) at 6 months, and 57.00 m (95% CI 7.83 to 106.16) at 12 months, again with I² = 0%. These findings suggest that the observed improvement in exercise capacity was generally consistent and was not driven solely by inclusion of the randomized trial, although the predominance of non-randomized studies should be considered when interpreting the magnitude of effect. (Fig. 4) (Fig. 5).

#### KCCQ overall score: (heart health)

**Fig. 6:**
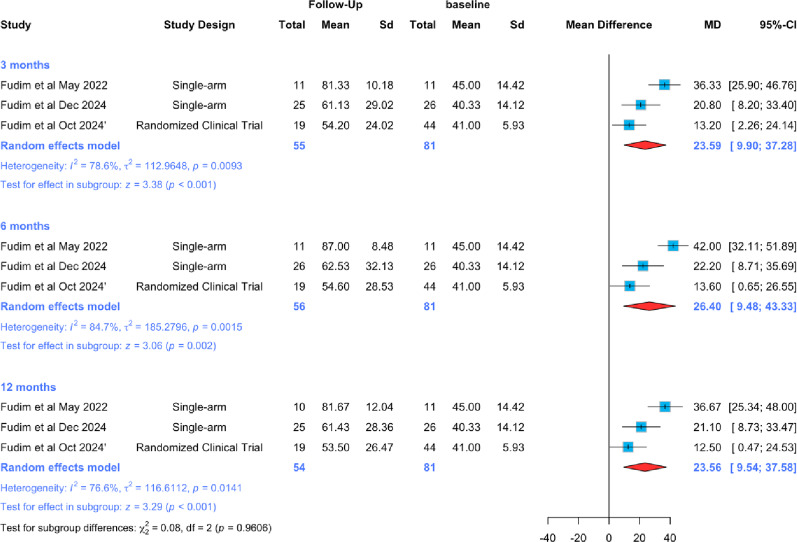
Forest plot of pooled effects for Kansas City Cardiomyopathy Questionnaire (KCCQ) overall score at 3, 6, and 12 months including all available studies

**Fig. 7:**
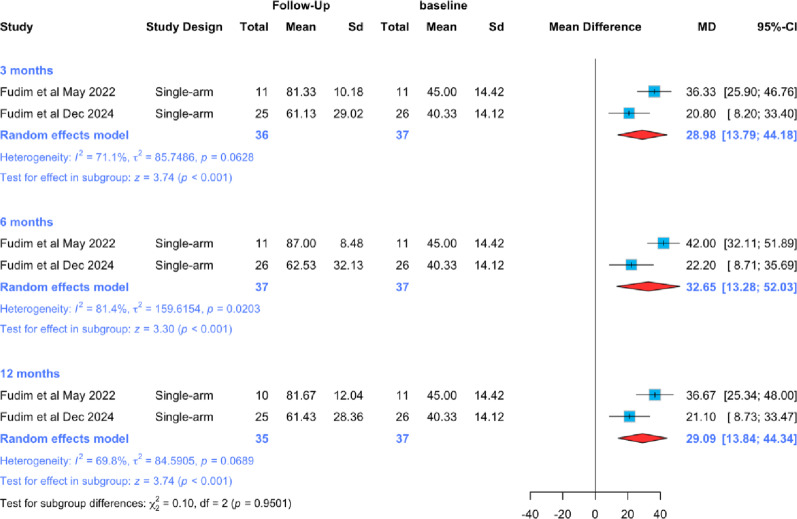
Sensitivity-analysis forest plot of pooled effects for Kansas City Cardiomyopathy Questionnaire (KCCQ) overall score at 3, 6, and 12 months after excluding the randomized controlled trial

When all available studies were included, pooled analysis showed a significant improvement in KCCQ overall score at 3, 6, and 12 months. The pooled mean difference was 23.59 points (95% CI 9.90 to 37.28) at 3 months, 26.40 points (95% CI 9.48 to 43.33) at 6 months, and 23.56 points (95% CI 9.54 to 37.58) at 12 months. Heterogeneity was substantial at all timepoints (I² = 78.6%, 84.7%, and 76.6%, respectively). In the sensitivity analysis excluding the randomized controlled trial, the direction and magnitude of effect remained similar. The pooled mean difference was 28.98 points (95% CI 13.79 to 44.18) at 3 months, 32.65 points (95% CI 13.28 to 52.03) at 6 months, and 29.09 points (95% CI 13.84 to 44.34) at 12 months, with persistent moderate-to-high heterogeneity (I² = 71.1%, 81.4%, and 69.8%, respectively). These findings suggest that the observed improvement in patient-reported health status was consistent and was not driven solely by inclusion of the randomized trial, although the substantial heterogeneity and predominance of non-randomized evidence warrant cautious interpretation. (Fig. 6) (Fig. 7).

### Biomarker and renal outcomes

#### NT-proBNP

**Fig. 8:**
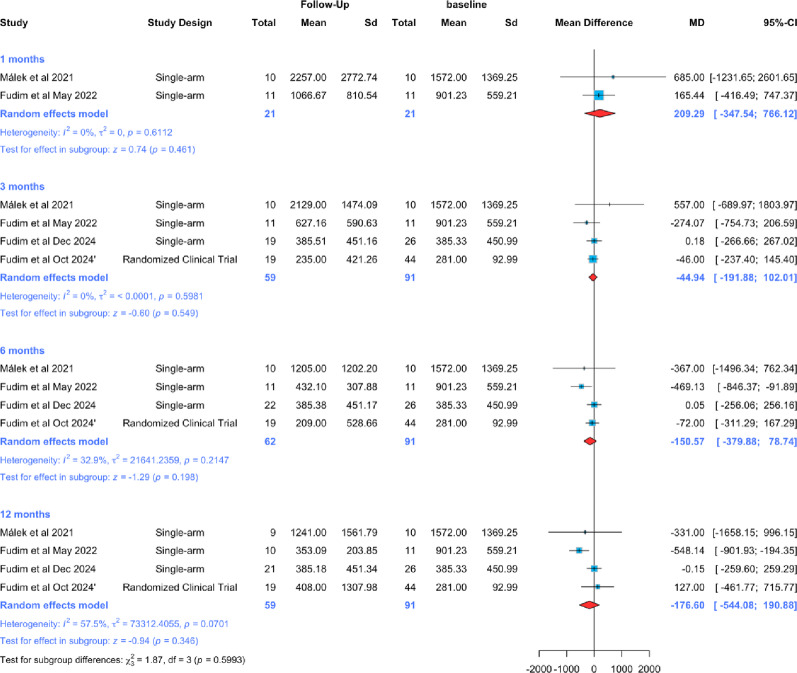
Forest plot of pooled effects for NT-proBNP (pg/mL) at 1, 3, 6, and 12 months including all available studies

**Fig. 9:**
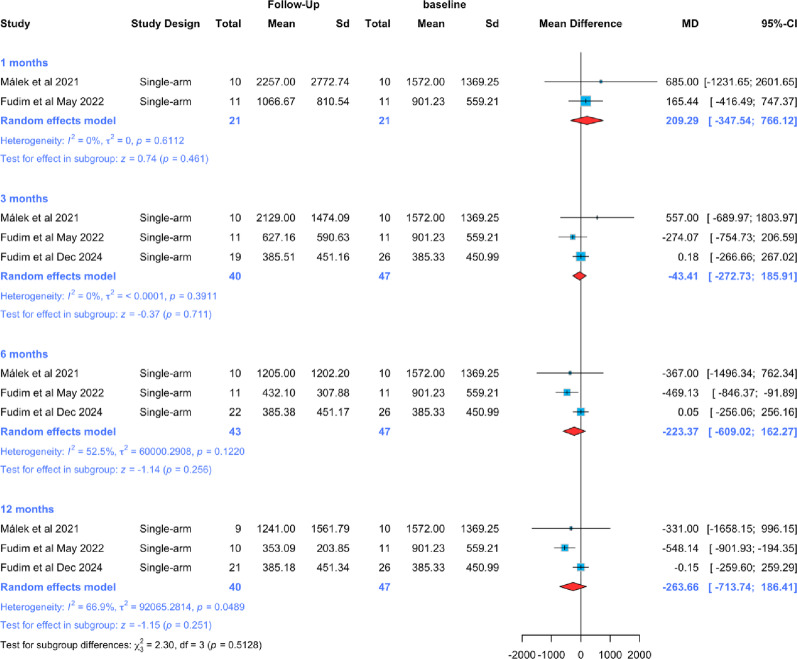
Sensitivity-analysis forest plot of pooled effects for NT-proBNP (pg/mL) at 1, 3, 6, and 12 months after excluding the randomized controlled trial

When all available studies were included, pooled analysis showed no statistically significant change in NT-proBNP at any assessed follow-up timepoint. The pooled mean difference was 209.29 pg/mL (95% CI − 347.54 to 766.12) at 1 month, − 44.94 pg/mL (95% CI − 191.88 to 102.01) at 3 months, − 150.57 pg/mL (95% CI − 379.88 to 78.74) at 6 months, and − 176.60 pg/mL (95% CI − 544.08 to 190.88) at 12 months. Heterogeneity was low at 1 month and 3 months (I² = 0%) but increased to 32.9% at 6 months and 57.5% at 12 months. In the sensitivity analysis excluding the randomized controlled trial, the direction of effect remained unchanged, and NT-proBNP still showed no statistically significant pooled reduction at any timepoint. The pooled mean difference was 209.29 pg/mL (95% CI − 347.54 to 766.12) at 1 month, − 43.41 pg/mL (95% CI − 272.73 to 185.91) at 3 months, − 223.37 pg/mL (95% CI − 609.02 to 162.27) at 6 months, and − 263.66 pg/mL (95% CI − 713.74 to 186.41) at 12 months. These findings suggest that the overall result was consistent in sensitivity analysis and was not driven solely by inclusion of the randomized trial, although the marked variability and wide confidence intervals warrant cautious interpretation. (Fig. 8) (Fig. 9).

#### Creatinine level, mg dL

**Fig. 10:**
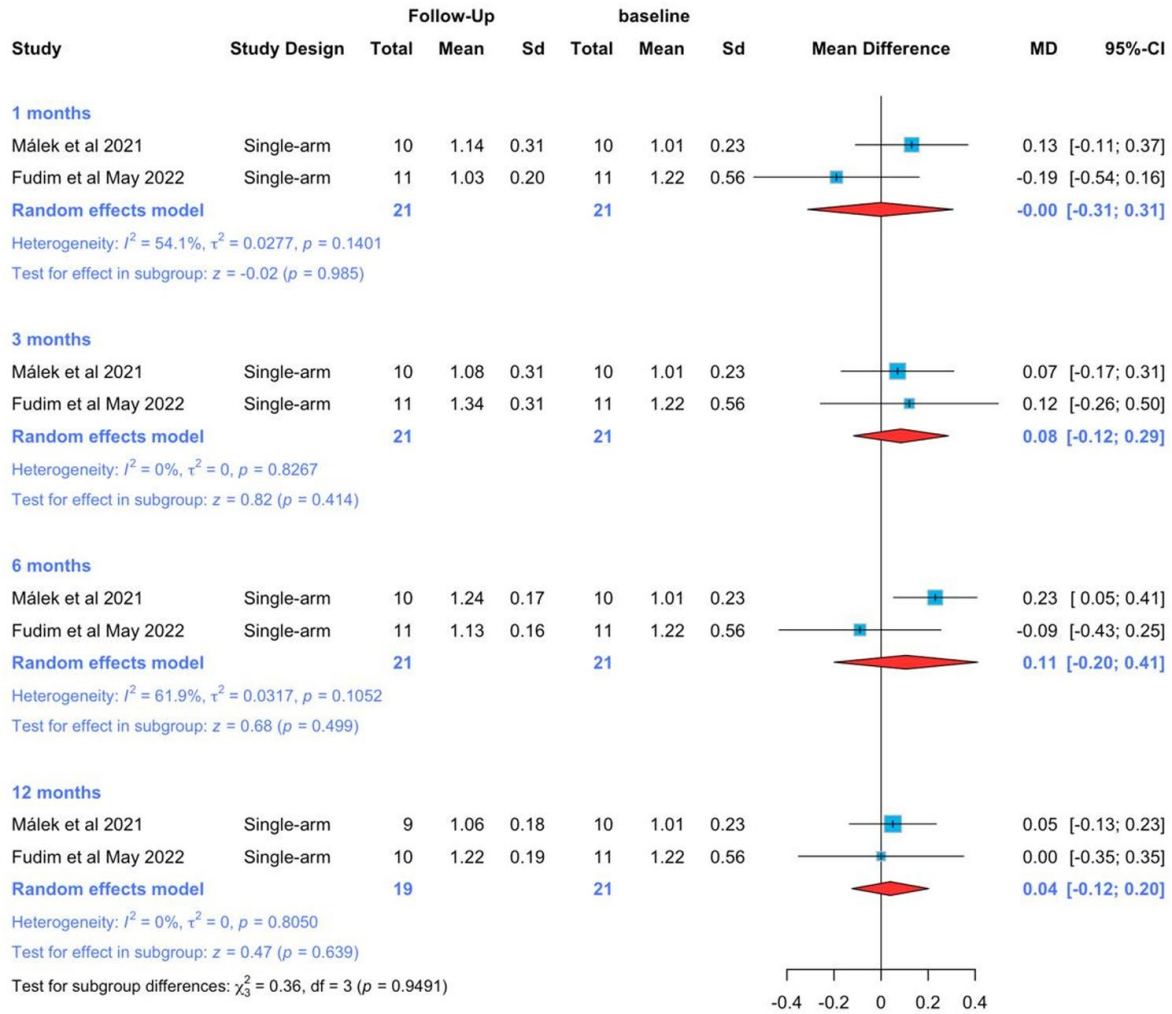
Forest plot showing the results for serum creatinine (mg/dL) at 1, 3, 6, and 12 months of follow-up. For studies reporting creatinine as median (interquartile range), values were converted to approximate mean and standard deviation using established conversion methods for exploratory pooling

Pooled analysis of uncontrolled studies showed no statistically significant change in serum creatinine at any assessed follow-up timepoint. The pooled mean difference was − 0.00 mg/dL (95% CI − 0.31 to 0.31) at 1 month, 0.08 mg/dL (95% CI − 0.12 to 0.29) at 3 months, 0.11 mg/dL (95% CI − 0.20 to 0.41) at 6 months, and 0.04 mg/dL (95% CI − 0.12 to 0.20) at 12 months. Heterogeneity was moderate at 1 month (I² = 54.1%) and 6 months (I² = 61.9%), but low at 3 months and 12 months (I² = 0%). Overall, these findings do not suggest a clear deterioration in renal function over follow-up. However, because this synthesis was derived from uncontrolled studies and included values converted from median (interquartile range) to approximate mean and standard deviation, the results should be interpreted cautiously. (Fig. 10) .

.

#### eGFR, mL min 1.73 m2

**Fig. 11:**
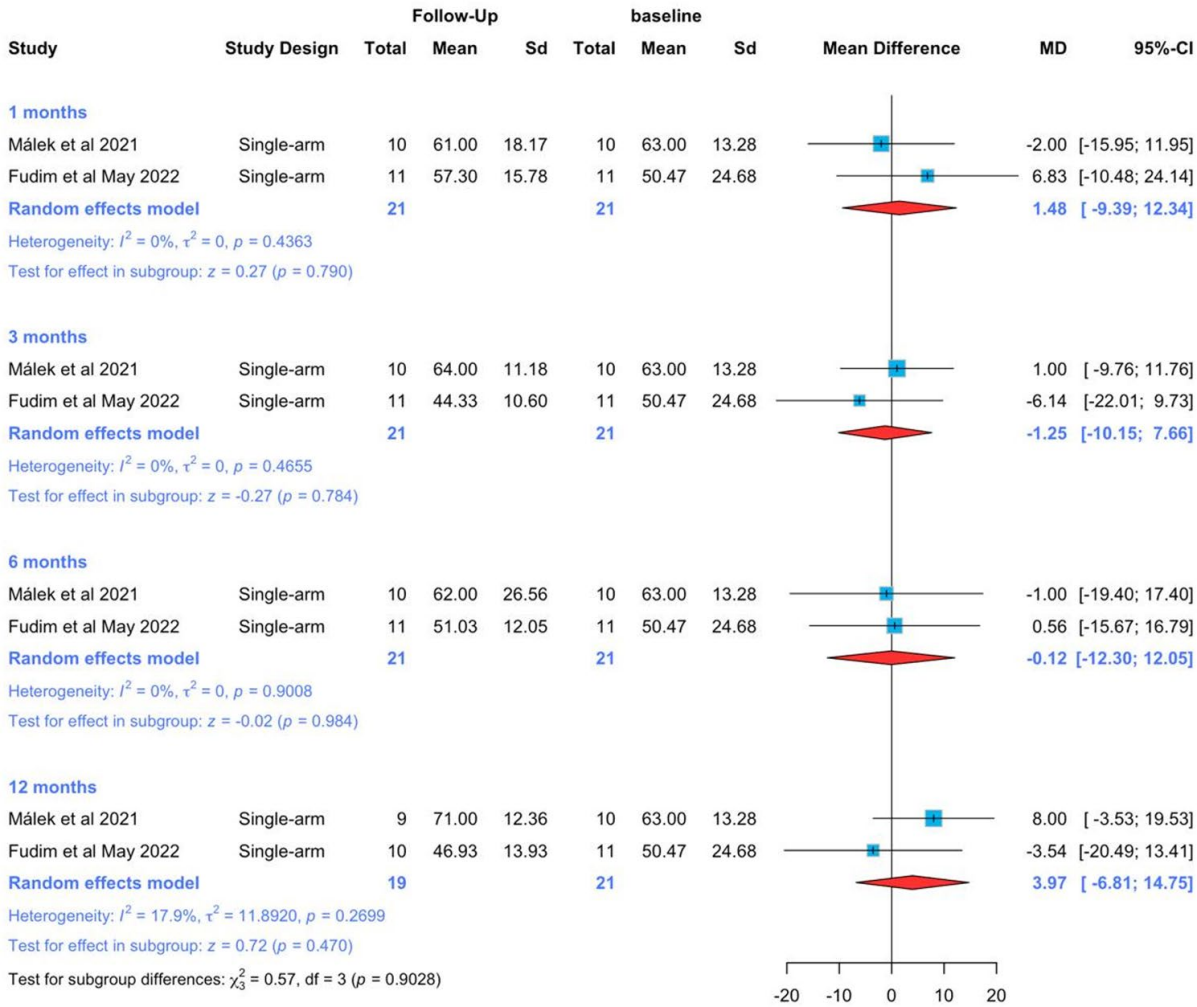
Forest plot showing the results for estimated glomerular filtration rate (eGFR, mL/min/1.73 m²) at 1, 3, 6, and 12 months of follow-up in uncontrolled studies

Pooled analysis of uncontrolled studies showed no statistically significant change in eGFR at any assessed follow-up timepoint. The pooled mean difference was 1.48 mL/min/1.73 m² (95% CI − 9.39 to 12.34) at 1 month, − 1.25 mL/min/1.73 m² (95% CI − 10.15 to 7.66) at 3 months, − 0.12 mL/min/1.73 m² (95% CI − 12.30 to 12.05) at 6 months, and 3.97 mL/min/1.73 m² (95% CI − 6.81 to 14.75) at 12 months. Heterogeneity was low at 1, 3, and 6 months (I² = 0%) and remained low at 12 months (I² = 17.9%). Overall, these findings do not suggest a clear change in renal function over follow-up. However, because this synthesis was derived from uncontrolled studies and included values converted from median (interquartile range) to approximate mean and standard deviation, the results should be interpreted cautiously (Fig. 11).

### Hemodynamic outcomes

#### ResLng PAP-S (mmHg)

**Fig. 12:**
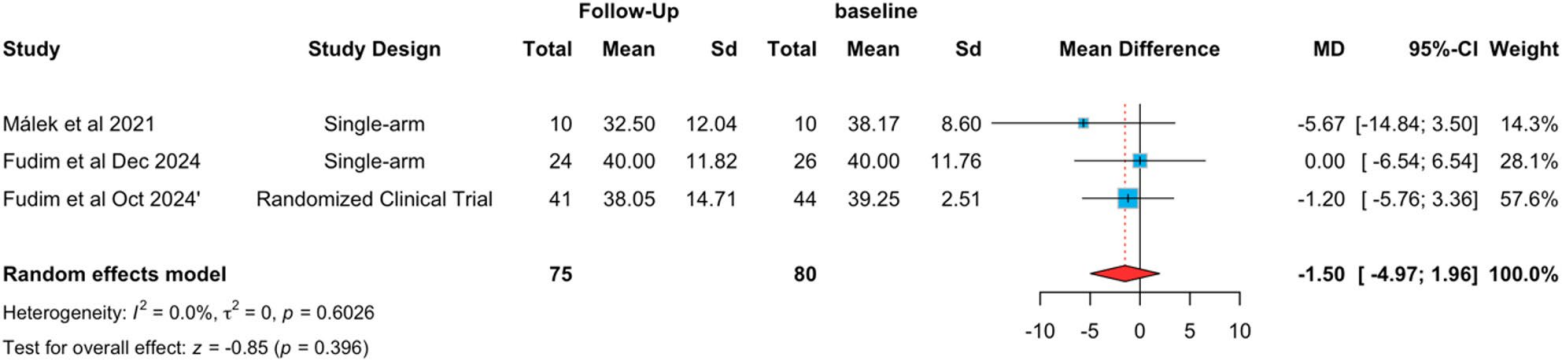
Forest plot of pooled effects for resting pulmonary artery systolic pressure (PAP-S, mmHg) at 1 month including all available studies

**Fig. 13:**
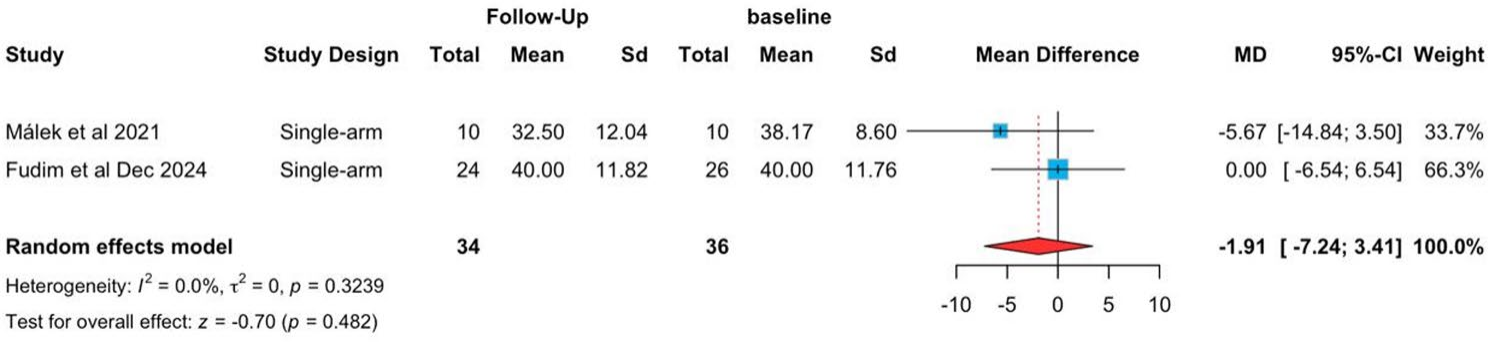
Sensitivity-analysis forest plot of pooled effects for resting pulmonary artery systolic pressure (PAP-S, mmHg) at 1 month after excluding the randomized controlled trial

When all available studies were included, pooled analysis showed no statistically significant change in resting pulmonary artery systolic pressure (PAP-S) at 1 month, with a pooled mean difference of − 1.50 mmHg (95% CI − 4.97 to 1.96). Heterogeneity was low (I² = 0%). In the sensitivity analysis excluding the randomized controlled trial, the result remained unchanged, with a pooled mean difference of − 1.91 mmHg (95% CI − 7.24 to 3.41), again with I² = 0%. These findings suggest that resting PAP-S did not significantly change over short-term follow-up, and this conclusion was not driven by inclusion of the randomized trial. However, because most included evidence was derived from uncontrolled studies, the result should still be interpreted cautiously (Fig. 12) (Fig. 13).

#### PCWP (mmHg)

##### Resting PCWP

**Fig. 14:**
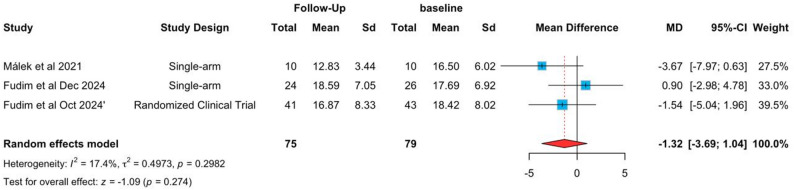
Forest plot of pooled effects for resting pulmonary capillary wedge pressure (PCWP, mmHg) at 1 month including all available studies

**Fig. 15:**
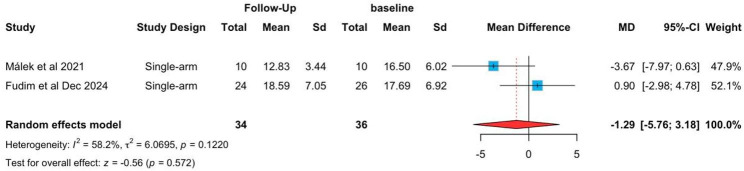
Sensitivity-analysis forest plot of pooled effects for resting pulmonary capillary wedge pressure (PCWP, mmHg) at 1 month after excluding the randomized controlled trial

When all available studies were included, pooled analysis showed no statistically significant change in resting pulmonary capillary wedge pressure (PCWP) at 1 month, with a pooled mean difference of − 1.32 mmHg (95% CI − 3.69 to 1.04). Heterogeneity was low (I² = 17.4%). In the sensitivity analysis excluding the randomized controlled trial, the result remained non-significant, with a pooled mean difference of − 1.29 mmHg (95% CI − 5.76 to 3.18), although heterogeneity was higher (I² = 58.2%). These findings suggest that resting PCWP did not significantly change at short-term follow-up, and this conclusion was not materially altered by inclusion of the randomized trial. However, because most included evidence was derived from uncontrolled studies, the result should be interpreted cautiously. (Fig. 14) (Fig. 15).

##### Leg-up PCWP

**Fig. 16:**
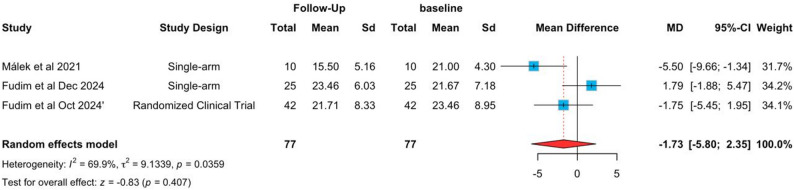
Forest plot of pooled effects for leg-up pulmonary capillary wedge pressure (PCWP, mmHg) at 1 month including all available studies

**Fig. 17:**
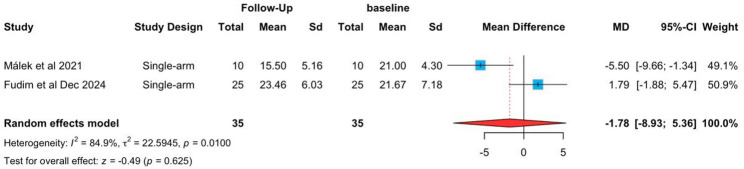
Sensitivity-analysis forest plot of pooled effects for leg-up pulmonary capillary wedge pressure (PCWP, mmHg) at 1 month after excluding the randomized controlled trial

When all available studies were included, pooled analysis showed no statistically significant change in leg-up pulmonary capillary wedge pressure (PCWP) at 1 month, with a pooled mean difference of − 1.73 mmHg (95% CI − 5.80 to 2.35). Heterogeneity was substantial (I² = 69.9%). In the sensitivity analysis excluding the randomized controlled trial, the result remained non-significant, with a pooled mean difference of − 1.78 mmHg (95% CI − 8.93 to 5.36), with considerable heterogeneity (I² = 84.9%). These findings suggest that leg-up PCWP did not significantly change at short-term follow-up, and this conclusion was not materially altered by inclusion of the randomized trial. However, the substantial heterogeneity and predominance of uncontrolled studies warrant cautious interpretation. (Fig. 16) (Fig. 17).

##### W PCWP

**Fig. 18:**
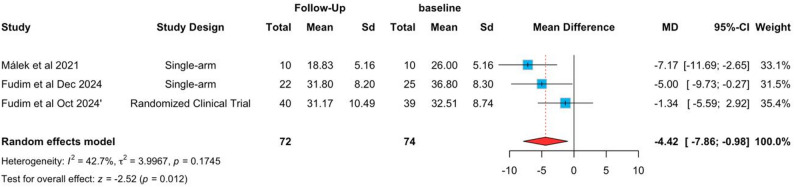
Forest plot of pooled effects for 20 W pulmonary capillary wedge pressure (PCWP, mmHg) at 1 month including all available studies

**Fig. 19:**
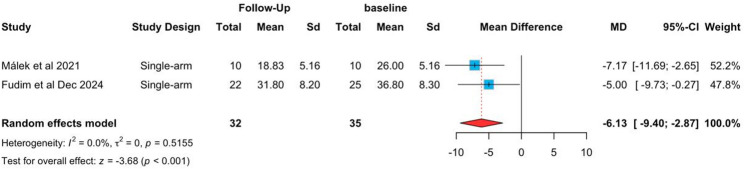
Sensitivity-analysis forest plot of pooled effects for 20 W pulmonary capillary wedge pressure (PCWP, mmHg) at 1 month after excluding the randomized controlled trial

When all available studies were included, pooled analysis showed a statistically significant reduction in 20 W pulmonary capillary wedge pressure (PCWP) at 1 month, with a pooled mean difference of − 4.42 mmHg (95% CI − 7.86 to − 0.98). Heterogeneity was moderate (I² = 42.7%). In the sensitivity analysis excluding the randomized controlled trial, the result remained significant, with a pooled mean difference of − 6.13 mmHg (95% CI − 9.40 to − 2.87), with no observed heterogeneity (I² = 0%). These findings suggest that 20 W PCWP improved at short-term follow-up, and this effect was preserved after exclusion of the randomized trial. However, because much of the evidence was derived from uncontrolled studies, the magnitude of benefit should be interpreted cautiously. (Fig. 18) (Fig. 19).

##### Peak PCWP

**Fig. 20:**
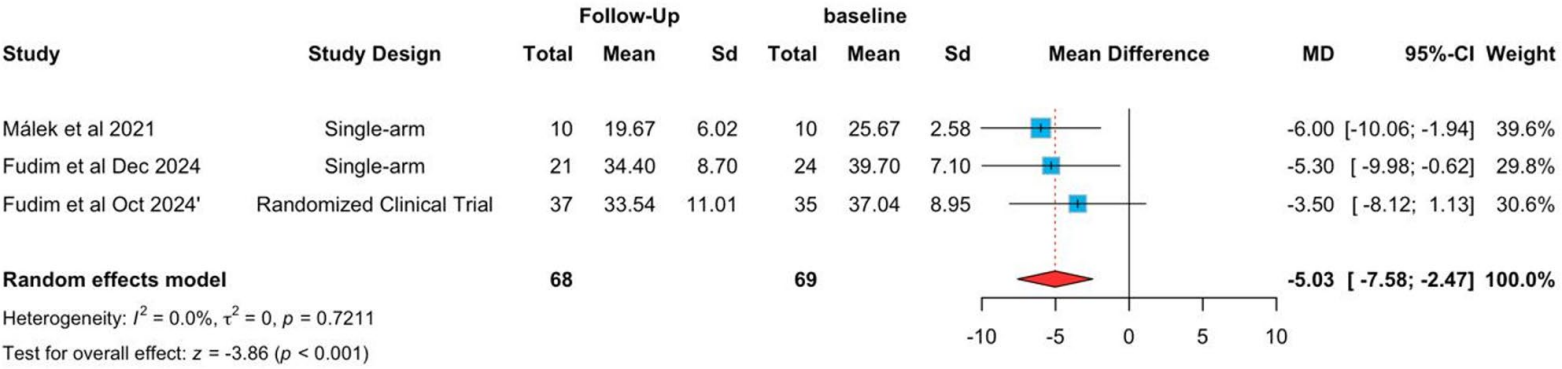
Forest plot of pooled effects for peak pulmonary capillary wedge pressure (PCWP, mmHg) at 1 month including all available studies

**Fig. 21:**
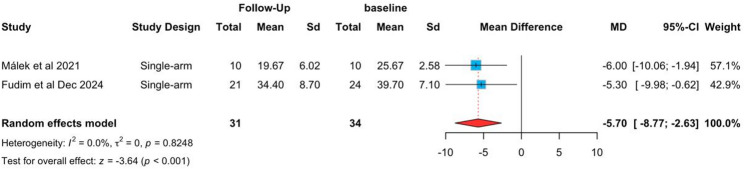
Sensitivity-analysis forest plot of pooled effects for peak pulmonary capillary wedge pressure (PCWP, mmHg) at 1 month after excluding the randomized controlled trial

Uncontrolled data suggested a reduction in peak pulmonary capillary wedge pressure (PCWP) after treatment. In Fudim 2024 lead-in, peak PCWP decreased from 39.7 ± 7.1 mmHg at baseline to 34.4 ± 8.7 mmHg at 30 days. In Málek et al. 2021, peak PCWP, reported as median (IQR), also decreased over follow-up. These uncontrolled findings should be interpreted cautiously because the synthesis combined directly reported mean-based values with converted median-based data. In the randomized sham-controlled trial, both groups showed numerical reductions in peak PCWP at 1 month, but no directly extractable comparative variance estimate was available; therefore, no formal randomized forest plot was generated for this endpoint. (Fig. 20) (Fig. 21).

##### Work-indexed peak PCWP

**Fig. 22:**
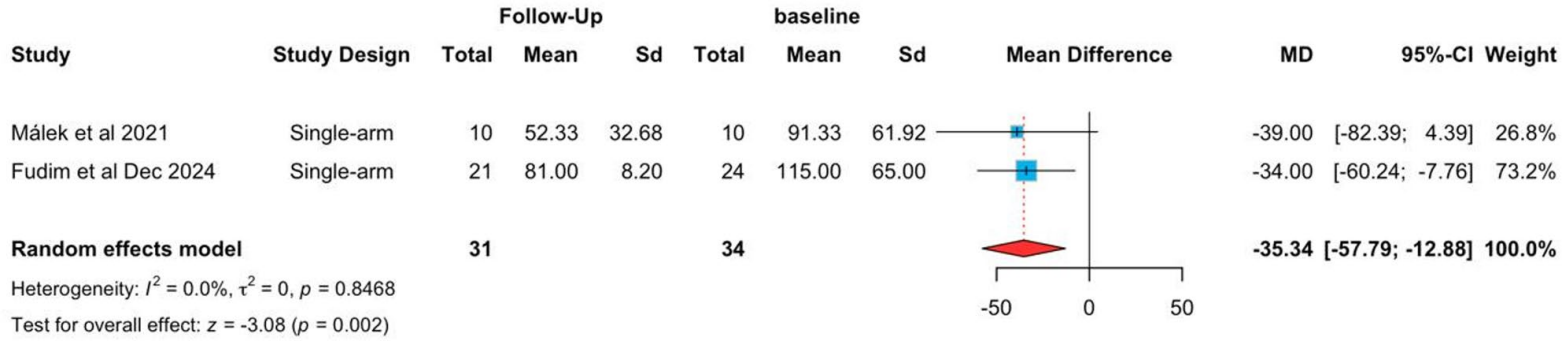
Forest plot showing the results for work-indexed peak pulmonary capillary wedge pressure (PCWP, mmHg/W/kg) at 1 month of follow-up in uncontrolled studies

Pooled analysis of uncontrolled studies showed a statistically significant reduction in work-indexed peak pulmonary capillary wedge pressure (PCWP) at 1 month, with a pooled mean difference of − 35.34 mmHg/W/kg (95% CI − 57.79 to − 12.88). Heterogeneity was low (I² = 0%). These findings suggest an improvement in workload-corrected peak filling pressures at short-term follow-up. However, because this synthesis was derived from uncontrolled studies and included values converted from median (interquartile range) to approximate mean and standard deviation for at least one study, the results should be interpreted cautiously. (Fig. 22)

#### Resting DBP (mmHg)

**Fig. 23:**
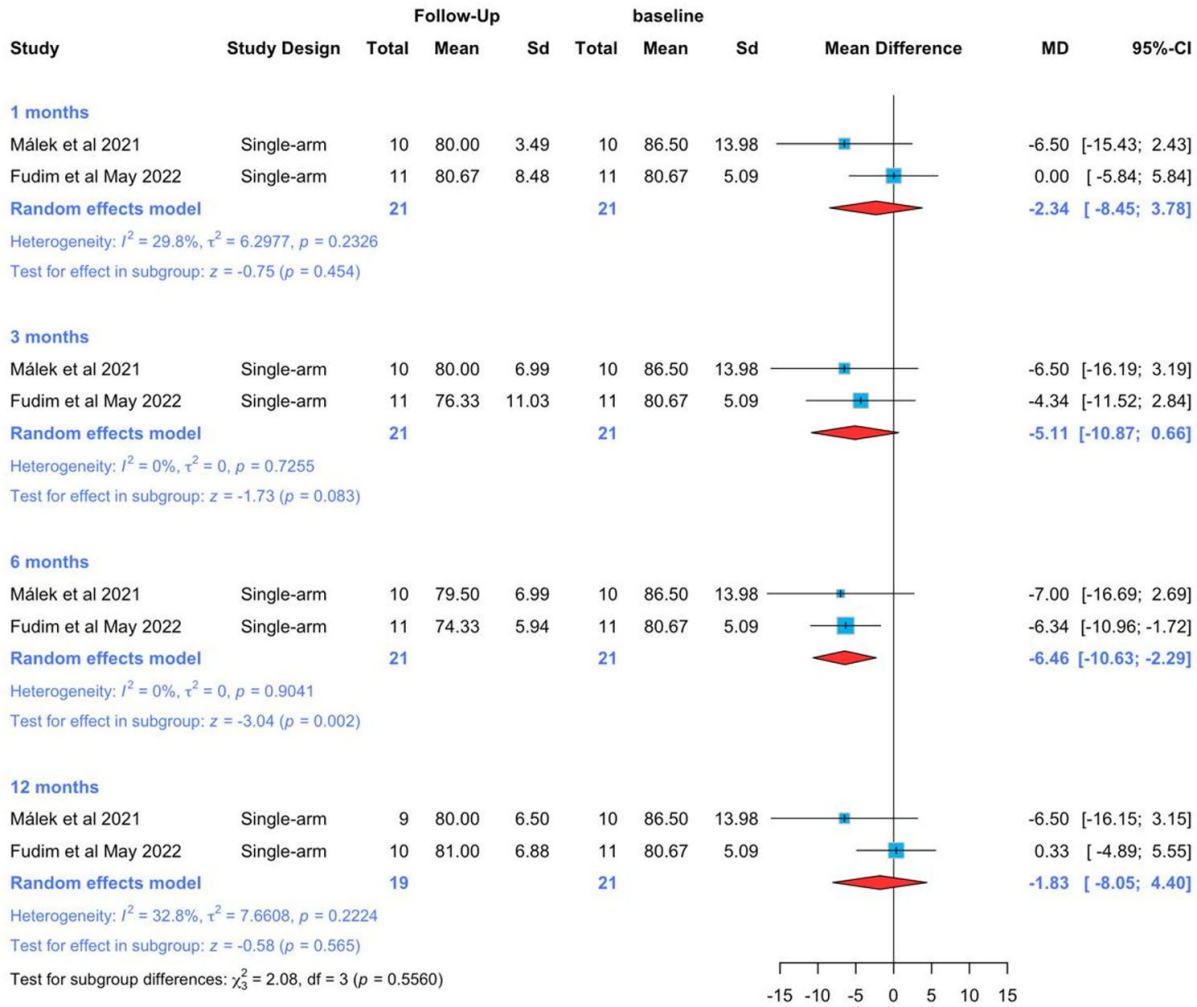
Forest plot showing the results for resting diastolic blood pressure (DBP, mmHg) at 1, 3, 6, and 12 months of follow-up in uncontrolled studies

Pooled analysis of uncontrolled studies showed no statistically significant change in resting diastolic blood pressure (DBP) at 1 month, 3 months, or 12 months. The pooled mean difference was − 2.34 mmHg (95% CI − 8.45 to 3.78) at 1 month, − 5.11 mmHg (95% CI − 10.87 to 0.66) at 3 months, and − 1.83 mmHg (95% CI − 8.05 to 4.40) at 12 months. A statistically significant reduction was observed at 6 months, with a pooled mean difference of − 6.46 mmHg (95% CI − 10.63 to − 2.29). Heterogeneity was low at 3 months and 6 months (I² = 0%), moderate at 1 month (I² = 29.8%), and moderate at 12 months (I² = 32.8%). Overall, these findings suggest that resting DBP was generally stable over follow-up, with a significant reduction observed only at 6 months. Because this synthesis was derived from uncontrolled studies, the findings should be interpreted cautiously. (Fig. 23)

#### Resting SBP (mmHg)

**Fig. 24:**
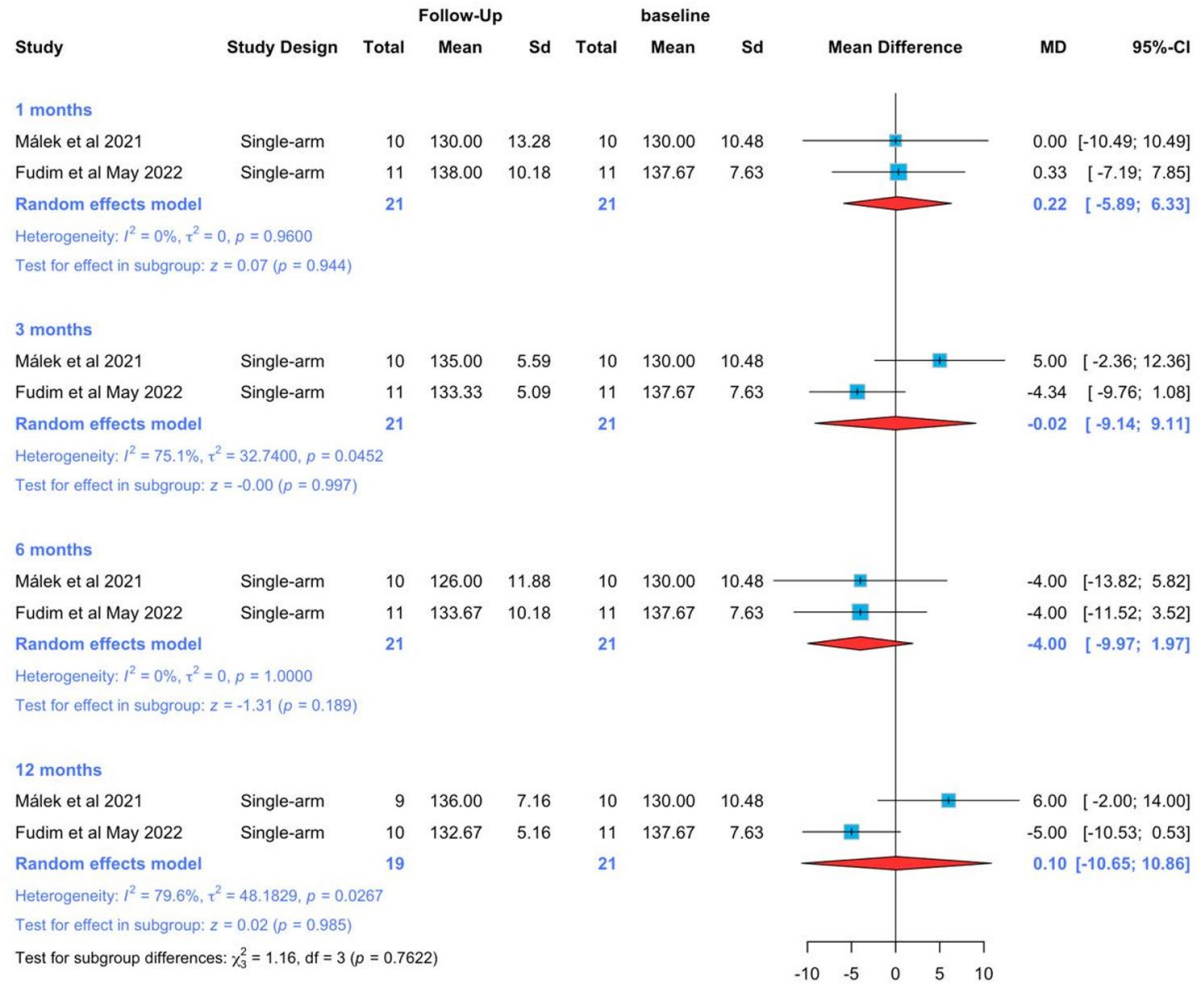
Forest plot showing the results for resting systolic blood pressure (SBP, mmHg) at 1, 3, 6, and 12 months of follow-up in uncontrolled studies

Pooled analysis of uncontrolled studies showed no statistically significant change in resting systolic blood pressure (SBP) at any assessed follow-up timepoint. The pooled mean difference was 0.22 mmHg (95% CI − 5.89 to 6.33) at 1 month, − 0.02 mmHg (95% CI − 9.14 to 9.11) at 3 months, − 4.00 mmHg (95% CI − 9.97 to 1.97) at 6 months, and 0.10 mmHg (95% CI − 10.65 to 10.86) at 12 months. Heterogeneity was low at 1 month and 6 months (I² = 0%), but substantial at 3 months (I² = 75.1%) and 12 months (I² = 79.6%). Overall, these findings suggest that resting SBP remained generally stable over follow-up. Because this synthesis was derived from uncontrolled studies, the results should be interpreted cautiously. (Fig. 24)

##### Resting HR (bpm)

**Fig. 25:**
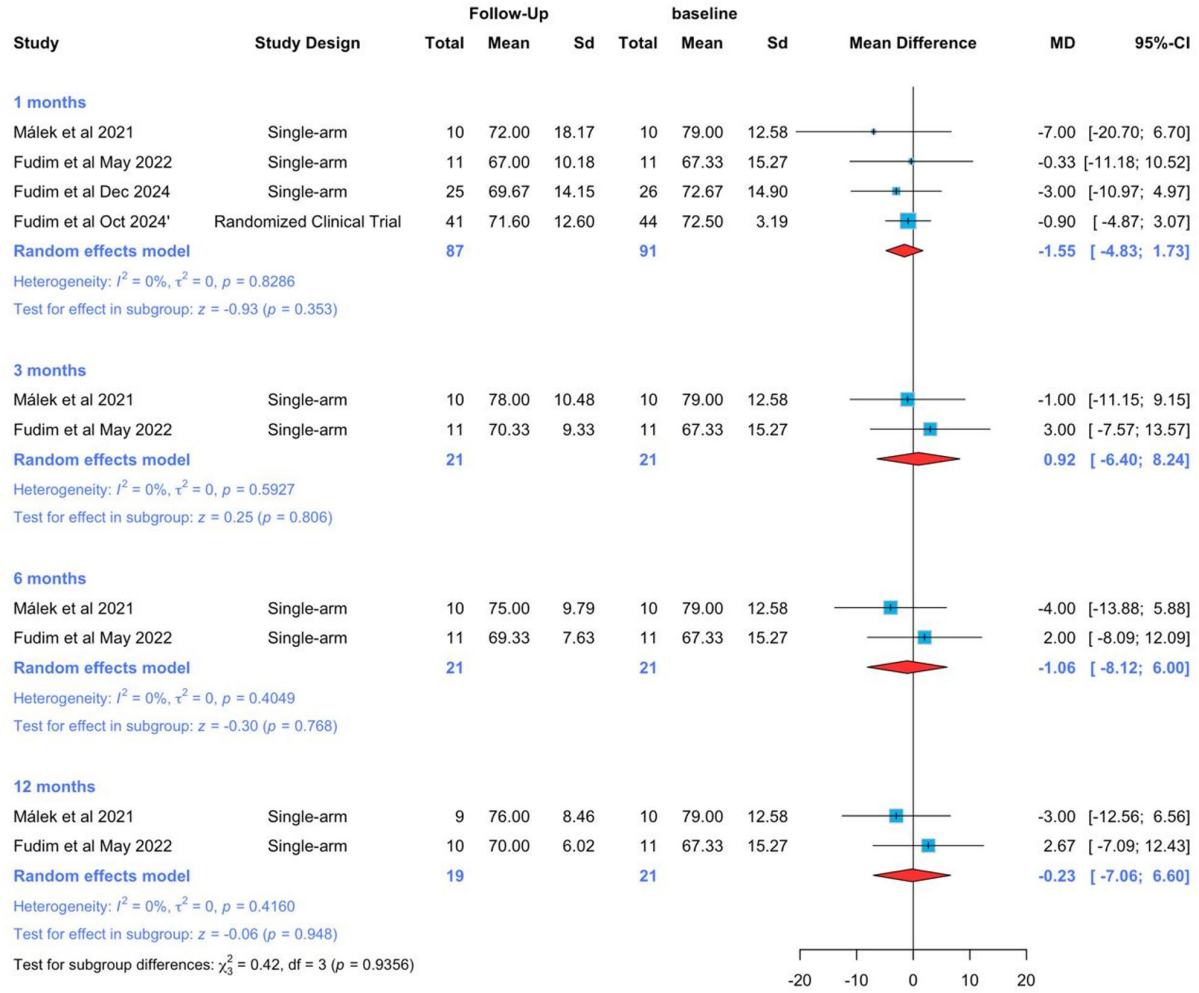
Forest plot of pooled effects for resting heart rate (HR, bpm) at 1, 3, 6, and 12 months including all available studies

**Fig. 26:**
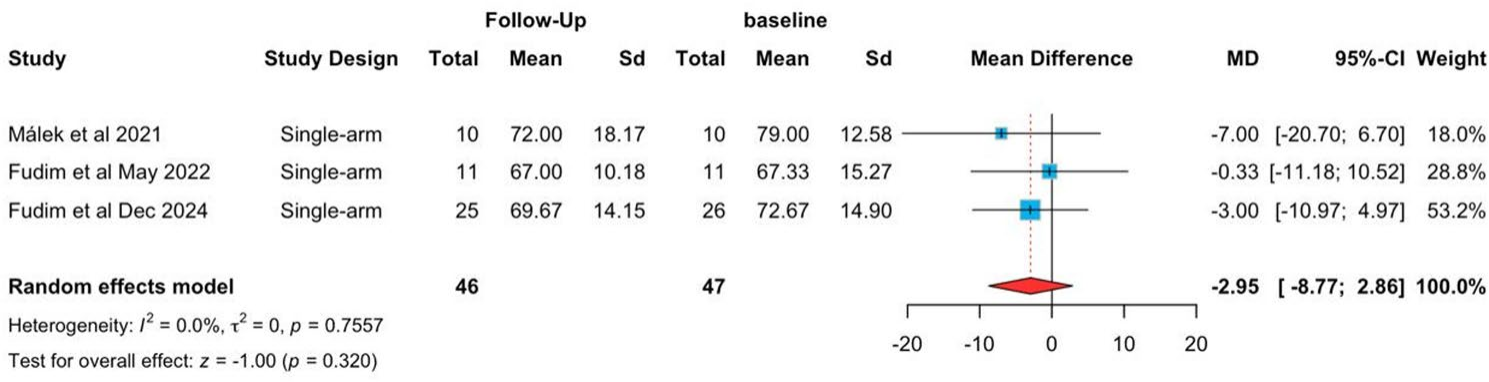
Sensitivity-analysis forest plot of pooled effects for resting heart rate (HR, bpm) at 1 month after excluding the randomized controlled trial

When all available studies were included, pooled analysis showed no statistically significant change in resting heart rate (HR) at any assessed follow-up timepoint. The pooled mean difference was − 1.55 bpm (95% CI − 4.83 to 1.73) at 1 month, 0.92 bpm (95% CI − 6.40 to 8.24) at 3 months, − 1.06 bpm (95% CI − 8.12 to 6.00) at 6 months, and − 0.23 bpm (95% CI − 7.06 to 6.60) at 12 months. Heterogeneity was low at all timepoints (I² = 0%). In the sensitivity analysis excluding the randomized controlled trial, the result at 1 month remained non-significant, with a pooled mean difference of − 2.95 bpm (95% CI − 8.77 to 2.86), again with I² = 0%. These findings suggest that resting HR remained generally stable over follow-up, and this conclusion was not materially altered by inclusion of the randomized trial. However, because most included evidence was derived from uncontrolled studies, the findings should be interpreted cautiously. .(Fig. 25) (Fig. 26).

#### LVEF (%)

**Fig. 27:**
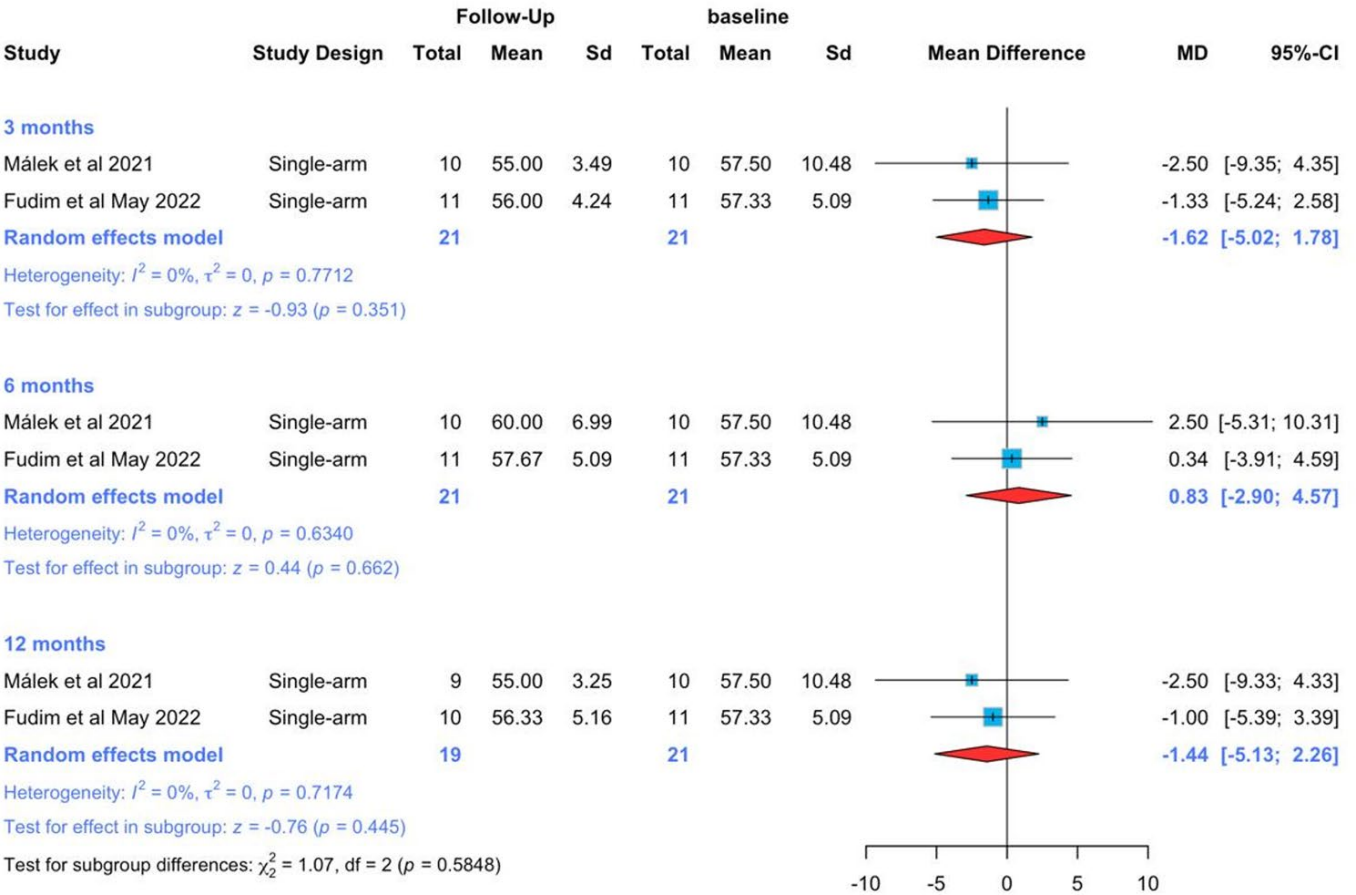
Forest plot showing the results for left ventricular ejection fraction (LVEF, %) at 3, 6, and 12 months of follow-up in uncontrolled studies

Pooled analysis of uncontrolled studies showed no statistically significant change in left ventricular ejection fraction (LVEF) at any assessed follow-up timepoint. The pooled mean difference was − 1.62% (95% CI − 5.02 to 1.78) at 3 months, 0.83% (95% CI − 2.90 to 4.57) at 6 months, and − 1.44% (95% CI − 5.13 to 2.26) at 12 months. Heterogeneity was low at all timepoints (I² = 0%). Overall, these findings suggest that LVEF remained generally stable over follow-up. Because this synthesis was derived from uncontrolled studies, the results should be interpreted cautiously (Fig. 27).

#### E/E′ septal

**Fig. 28:**
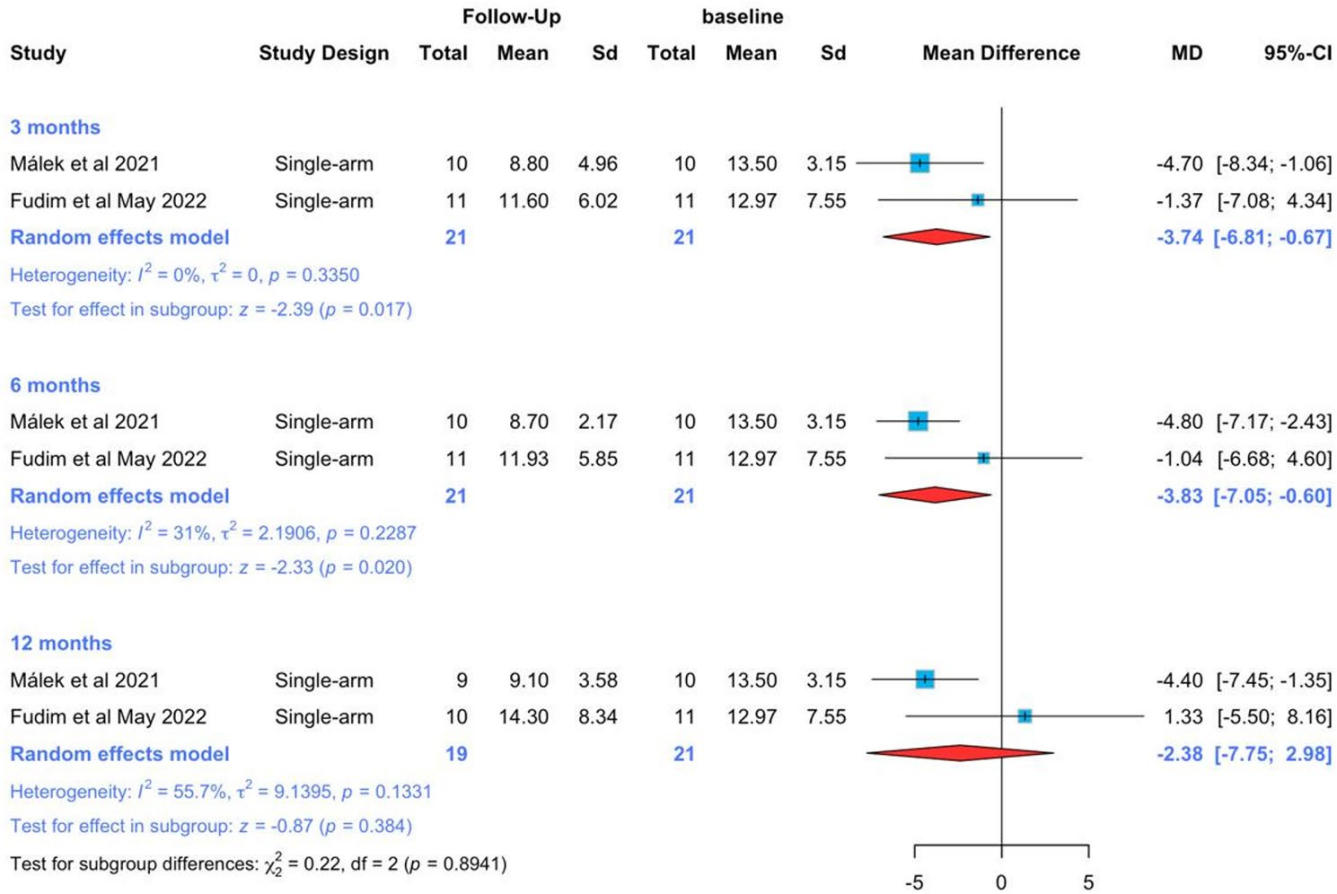
Forest plot showing the results for E/E′ septal at 3, 6, and 12 months of follow-up in uncontrolled studies

Pooled analysis of uncontrolled studies showed a statistically significant reduction in E/E′ septal at 3 months and 6 months, but not at 12 months. The pooled mean difference was − 3.74 (95% CI − 6.81 to − 0.67) at 3 months, − 3.83 (95% CI − 7.05 to − 0.60) at 6 months, and − 2.38 (95% CI − 7.75 to 2.98) at 12 months. Heterogeneity was low at 3 months (I² = 0%), moderate at 6 months (I² = 31%), and moderate at 12 months (I² = 55.7%). Overall, these findings suggest improvement in diastolic filling pressure at intermediate follow-up, although the effect was not sustained statistically at 12 months. Because this synthesis was derived from uncontrolled studies, the results should be interpreted cautiously (Fig. 28).

.

**Table 5:** Summary of pooled clinical, functional, and hemodynamic outcomes

Outcome	Main finding
6-minute walk test (6MWT)	Improved over follow-up; results remained directionally similar after excluding the randomized controlled trial
KCCQ overall score	Improved over follow-up; findings were preserved in sensitivity analysis, although heterogeneity was substantial
Resting PAP-S	No significant pooled change at 1 month
Resting PCWP	No significant pooled change at 1 month
Leg-up PCWP	No significant pooled change at 1 month
20 W PCWP	Significant reduction at 1 month; effect remained after excluding the randomized controlled trial
Peak PCWP	Significant reduction at 1 month; effect remained after excluding the randomized controlled trial
Work-indexed peak PCWP	Significant reduction at 1 month in uncontrolled studies

Table 5: This table provides a concise summary of the main pooled clinical, functional, and hemodynamic findings. For selected outcomes, pooled analyses including all available studies were followed by sensitivity analyses excluding the randomized controlled trial. Abbreviations: 6MWT, 6-minute walk test; KCCQ, Kansas City Cardiomyopathy Questionnaire; PAP-S, pulmonary artery systolic pressure; PCWP, pulmonary capillary wedge pressure.

**Table 6:** Summary of biomarker, renal, echocardiographic, and safety outcomes

Outcome	Main finding
NT-proBNP	No significant pooled change over follow-up; similar findings after excluding the randomized controlled trial
Serum creatinine	No clear overall change over follow-up
eGFR	No clear overall change over follow-up
Resting DBP	Mostly stable over follow-up; significant reduction at 6 months only
Resting SBP	No significant pooled change over follow-up
Resting HR	No significant pooled change over follow-up; similar findings in sensitivity analysis
LVEF	No significant pooled change over follow-up
E/E′ septal	Significant reduction at 3 and 6 months; not significant at 12 months
Safety/adverse events	Reported descriptively across studies; no consistent pooled safety signal established

Table 6: This table provides a concise summary of biomarker, renal, echocardiographic, and safety findings. Several analyses were based on uncontrolled studies, and some outcomes required conversion of median and interquartile range to approximate mean and standard deviation for exploratory synthesis. Abbreviations: NT-proBNP, N-terminal pro-B-type natriuretic peptide; eGFR, estimated glomerular filtration rate; DBP, diastolic blood pressure; SBP, systolic blood pressure; HR, heart rate; LVEF, left ventricular ejection fraction.

## Discussion

The greater splanchnic nerve plays an important role in cardiovascular homeostasis through regulation of splanchnic vascular tone and capacitance. In heart failure, heightened sympathetic activation reduces splanchnic venous capacitance and promotes transfer of blood from the abdominal compartment to the thoracic circulation, thereby increasing cardiac filling pressures and worsening pulmonary congestion, particularly during exercise or stress [9, 10]. In HFpEF, where impaired diastolic reserve and ventricular stiffness predispose patients to marked rises in filling pressure, this mechanism provides a biologically plausible rationale for targeting the right GSN [7–10]. Early surgical and transvenous studies suggested that interruption of right GSN activity may reduce exercise-related intracardiac filling pressures and improve symptoms in selected patients with HFpEF [11–15].

In the present systematic review and meta-analysis, right GSN ablation was associated with favorable changes in several functional, hemodynamic, and patient-reported outcomes. However, these findings must be interpreted cautiously. Much of the apparent benefit was derived from small uncontrolled studies reporting pre-post changes from baseline, whereas randomized sham-controlled evidence did not demonstrate clear comparative benefit across several key endpoints. Accordingly, the available evidence should be viewed as suggestive rather than definitive, and pooled baseline-to-follow-up improvements should not be interpreted as equivalent to proof of treatment efficacy in a comparative sense [14–16].

### Functional capacity and quality of life

The clearest clinical signal in this review was improvement in functional capacity and patient-reported health status, particularly for 6-minute walk test (6MWT) distance and Kansas City Cardiomyopathy Questionnaire (KCCQ) score. These outcomes are clinically meaningful because reduced exercise tolerance and impaired quality of life are central features of HFpEF and major determinants of patient burden [1, 2, 7, 8]. The observed improvements are also biologically plausible, as attenuation of stress-related congestion could be expected to improve exertional symptoms and daily functional status [9, 10].At the same time, these outcomes are especially vulnerable to expectancy effects, regression to the mean, selective retention of responders, and differences in follow-up intensity when derived predominantly from uncontrolled cohorts [16–19]. This concern is particularly relevant for KCCQ, for which heterogeneity was moderate to high across several pooled analyses. Importantly, the randomized sham-controlled evidence did not confirm a clear comparative benefit for either 6MWT or KCCQ in an unselected HFpEF population [16]. Therefore, the observed improvements in 6MWT and KCCQ should be interpreted as signals of potential benefit that support further investigation rather than as established therapeutic effects [16–19].

### Hemodynamic outcomes

Among the hemodynamic outcomes, the most mechanistically coherent findings were seen in exercise-related or provocative filling pressure measures, particularly 20 W PCWP and peak PCWP. This pattern is consistent with the proposed mechanism of GSN ablation, namely increasing splanchnic capacitance and reducing pathologic central volume shift during physiologic stress [7, 9, 10]. Early physiologic and interventional studies reported reductions in exercise PCWP shortly after right GSN interruption, supporting this concept [11–15]. Likewise, the reduction observed in E/E′ septal in uncontrolled studies may reflect improved diastolic filling dynamics and reduced congestion, although this finding remains exploratory.In contrast, resting PCWP, leg-up PCWP, and resting PAP-S were not consistently improved. This is also physiologically plausible, because the splanchnic reservoir may be particularly relevant during exertion rather than under resting conditions [7, 9, 10]. However, the overall hemodynamic evidence remains mixed. The sham-controlled REBALANCE-HF trial did not confirm significant comparative benefit in the primary exercise-related hemodynamic endpoint, and directly extractable comparative estimates were not available for every provocative subtype [16]. Taken together, the current data suggest that any hemodynamic benefit may be context-dependent, phenotype-specific, or more apparent during exercise than at rest [16–19].

### Secondary and neutral outcomes

Several secondary outcomes were largely neutral, including NT-proBNP, resting pulmonary artery systolic pressure, resting heart rate, resting systolic blood pressure, most resting diastolic blood pressure analyses, renal indices, and left ventricular ejection fraction (LVEF). These findings suggest that any potential benefit of GSN ablation may relate more to dynamic congestion and symptom burden than to broad changes in resting biomarker profiles, systemic hemodynamics, or systolic function [2, 7, 9, 10]. The absence of a significant pooled effect on NT-proBNP is notable, because it argues against a consistent biomarker signal despite some favorable hemodynamic and functional findings.The relative stability of serum creatinine and eGFR is somewhat reassuring from a short-term renal perspective, although renal safety cannot be considered definitively established given the small number of studies, the predominance of uncontrolled evidence, and the need for conversion of some renal data from median-based reporting. Similarly, the absence of meaningful change in LVEF is not unexpected in HFpEF, where symptoms are often driven more by impaired diastolic reserve, ventricular-vascular interaction, and abnormal volume redistribution than by overt systolic dysfunction [1, 2, 7, 8, 17–19]. Overall, these neutral findings support the view that the intervention, if beneficial, is more likely to affect exercise-related physiology than resting cardiovascular structure or broad biomarker profiles.

### Interpretation of the evidence base

A major challenge in interpreting this literature is the discrepancy between encouraging early uncontrolled studies and the more neutral randomized evidence. Several factors may explain this difference. First, HFpEF is a heterogeneous syndrome, and not all patients are equally affected by splanchnic-mediated volume redistribution [2, 17–19]. Second, open-label and single-arm studies may preferentially identify responders and may amplify subjective or functional improvement through expectancy effects [16–19]. Third, benefits observed in exploratory cohorts may not be sufficiently durable or reproducible to generate clear between-group differences under sham-controlled conditions [15, 16]. Taken together, the current evidence does not support the conclusion that right GSN ablation has established efficacy across the general HFpEF population. A more appropriate interpretation is that benefit, if present, may be restricted to selected physiologic phenotypes, making responder identification and phenotype-guided trial design especially important [16–19].

### Strengths and limitations

This review synthesizes an emerging therapeutic strategy with a strong physiologic rationale in a population with limited treatment options [1, 2, 7–10]. Strengths of the present analysis include the clearer distinction between pooled analyses including all eligible studies and sensitivity analyses excluding the randomized controlled trial, explicit attention to possible overlapping publications, and cautious handling of outcomes reported in mixed formats. However, several limitations restrict interpretation. The total evidence base was small, and much of the favorable signal came from uncontrolled or exploratory studies that are inherently more susceptible to confounding, selection bias, regression to the mean, and placebo effects [16–19]. In addition, some quantitative syntheses required conversion of median and interquartile range to approximate mean and standard deviation, making these analyses exploratory by nature. Heterogeneity in study design procedural approach to right greater splanchnic nerve ablation, follow-up duration, outcome definitions, and reporting format also reduced confidence in several pooled estimates. Finally, publication bias cannot be excluded; given the small number of studies, funnel plot methods would have limited value and low power, so the absence of formal asymmetry testing would not exclude missing negative evidence [20, 21].

### Clinical implications and future directions

At present, endovascular ablation of the right greater splanchnic nerve should be regarded as an investigational strategy rather than an established adjunctive therapy for HFpEF. Its physiologic rationale is compelling, and early uncontrolled studies suggest possible benefit in selected hemodynamic and patient-reported outcomes [11–15]. However, the sham-controlled randomized evidence does not support broad adoption in an unselected HFpEF population [16]. These mixed findings are consistent with the broader concept that HFpEF is a heterogeneous syndrome in which mechanism-based therapies may only benefit selected phenotypes [17–19]. Future studies should therefore prioritize larger sham-controlled trials, clearer phenotype enrichment, separation of exercise-related from resting hemodynamic endpoints, longer follow-up, and direct evaluation of whether benefit is concentrated in patients with demonstrable exercise-induced congestion or other splanchnic-dominant hemodynamic profiles. It would also be valuable to study this intervention in the context of contemporary guideline-directed HFpEF therapy, including SGLT2 inhibitors [3–6].

### Conclusion

In summary, current evidence suggests that right GSN ablation has potential clinical relevance in HFpEF, particularly for improving selected exercise-related hemodynamic, functional, and patient-reported outcomes. The observed improvements in 6MWT, KCCQ, and some provocative PCWP measures support continued investigation of this strategy as a potentially important mechanism-based intervention. However, these findings are driven largely by small uncontrolled studies, whereas randomized sham-controlled evidence has not demonstrated consistent comparative efficacy in a broad HFpEF population [14–16]. Accordingly, right GSN ablation should currently be regarded as a promising investigational approach rather than an established adjunctive therapy. Further well-designed sham-controlled trials are needed to clarify its clinical role, identify the patients most likely to benefit, and determine whether the observed physiologic and symptomatic effects are reproducible and clinically meaningful within contemporary HFpEF care [16–19].

## Supplementary Information

Below is the link to the electronic supplementary material.

Supplementary Material 1.

## Data Availability

Data for the current study are available from the corresponding author.

## Abbreviations

6MWT: 6-Minute Walk Test
HFpEF: Heart failure with preserved ejection fraction
KCCQ: Kansas city cardiomyopathy questionnaire
PCWP: Pulmonary capillary wedge pressure
PAP: Pulmonary artery pressure
DBP: Diastolic blood pressure
E/E′: E/E′ septal ratio (indicative of diastolic function)
NT-proBNP: N-terminal pro B-type natriuretic peptide
eGFR: Estimated glomerular filtration rate
LVEF: Left ventricular ejection fraction
HR: Heart rate
SBP: Systolic blood pressure
MeSH: Medical subject headings
PICO: Population, intervention, comparison, outcome (used in evidence-based research)
PRISMA: Preferred reporting items for systematic reviews and meta-analyses
NYHA: New York Heart Association
