# Supplementary material for: Endovascular ablation of the right greater splanchnic nerve for the management of heart failure with preserved ejection fraction: a systematic review and meta-analysis
Source: Egypt Heart J. 2026 Jun 8;78:43. doi: 10.1186/s43044-026-00748-1 (PMC13246998; doi:10.1186/s43044-026-00748-1)
Supplement: Supplementary file 1 — Supplementary Material 1. [file 43044_2026_748_MOESM1_ESM.docx]

**Supplementary Document
Database Search Strategy and Search Documentation**

*For: Endovascular Ablation of the Right Greater Splanchnic Nerve in Heart Failure With Preserved Ejection Fraction: A Systematic Review and Meta-analysis*

# 1. Search overview

A systematic literature search was conducted in December 2024 to identify studies evaluating right greater splanchnic nerve ablation in patients with heart failure with preserved ejection fraction (HFpEF).

The primary bibliographic databases searched were PubMed, Scopus, and Embase. Google Scholar and ScienceDirect were used only as supplementary sources for manual screening and reference tracking rather than as primary databases for reproducible evidence retrieval.

CENTRAL and Web of Science were not included in the primary search. The review focused on PubMed, Scopus, and Embase because these databases were expected to capture the core biomedical and multidisciplinary literature for this small and emerging intervention-specific field. Nevertheless, omission of CENTRAL and Web of Science may have reduced search sensitivity and should be considered when interpreting search comprehensiveness.

Conference abstracts were excluded because they often lacked sufficient methodological detail, complete outcome reporting, and reliable information on cohort overlap. However, exclusion of conference abstracts may have increased the risk of publication bias in this emerging field.

# 2. Exact search dates and database yields

| Source | Records retrieved | Role in review |
| --- | --- | --- |
| PubMed | 15 | Primary database |
| Scopus | 17 | Primary database |
| Embase | 23 | Primary database |
| Google Scholar | 0 | Supplementary/manual screening only |
| ScienceDirect | 0 | Supplementary/manual screening only |
| Total before deduplication | 55 | Should match PRISMA exactly |

# 3. Search concepts and eligibility frame

- Population: patients with heart failure with preserved ejection fraction (HFpEF).
- Intervention: endovascular ablation of the right greater splanchnic nerve.
- Comparison: standard care, sham procedure, medical therapy, or no formal comparator depending on study design.
- Outcomes of interest:
- Primary outcomes
- Functional capacity, specifically 6-minute walk test (6MWT)
- Hemodynamic outcomes, specifically pulmonary capillary wedge pressure (PCWP)
- Patient-reported health status, specifically Kansas City Cardiomyopathy Questionnaire (KCCQ) overall score
- Secondary outcomes
- Biomarker outcomes, including NT-proBNP
- Renal outcomes, including serum creatinine and estimated glomerular filtration rate (eGFR)
- Echocardiographic and cardiac function parameters, including left ventricular ejection fraction (LVEF) and E/E′ septal
- Additional hemodynamic measures, including resting pulmonary artery systolic pressure (PAP-S), blood pressure, and heart rate
- Safety and clinical event outcomes, including hospitalization, adverse events, and mortality, where reported

# 4. Database-specific search strategies

The following strategies are presented in a reproducible format. Syntax may be adapted to the exact platform interface used at the time of the search.

## PubMed

("heart failure with preserved ejection fraction"[Title/Abstract] OR "HFpEF"[Title/Abstract] OR "heart failure with preserved EF"[Title/Abstract] OR "heart failure"[Title/Abstract] OR "HF"[Title/Abstract])
AND
("endovascular ablation"[Title/Abstract] OR "right greater splanchnic nerve ablation"[Title/Abstract] OR "splanchnic nerve denervation"[Title/Abstract] OR "splanchnic nerve ablation"[Title/Abstract] OR "greater splanchnic nerve"[Title/Abstract])

Records retrieved: 15

## Scopus

TITLE-ABS-KEY ( "heart failure with preserved ejection fraction" OR HFpEF OR "heart failure with preserved EF" OR "heart failure" OR HF )
AND
TITLE-ABS-KEY ( "endovascular ablation" OR "right greater splanchnic nerve ablation" OR "splanchnic nerve denervation" OR "splanchnic nerve ablation" OR "greater splanchnic nerve" )

Records retrieved: 17

## Embase

( "heart failure with preserved ejection fraction":ti,ab OR HFpEF:ti,ab OR "heart failure with preserved EF":ti,ab OR "heart failure":ti,ab OR HF:ti,ab )
AND
( "endovascular ablation":ti,ab OR "right greater splanchnic nerve ablation":ti,ab OR "splanchnic nerve denervation":ti,ab OR "splanchnic nerve ablation":ti,ab OR "greater splanchnic nerve":ti,ab )

Records retrieved: 23

## Google Scholar and ScienceDirect

Google Scholar and ScienceDirect were used only as supplementary sources for manual screening and backward/forward reference tracking. They were not treated as primary reproducible bibliographic databases in the formal quantitative search strategy.
